# Supplementary material for: Genetically Defined Functional Modules for Spatial Orienting in the Mouse Superior Colliculus
Source: Curr Biol. 2019 Sep 9;29(17):2892–2904.e8. doi: 10.1016/j.cub.2019.07.083 (PMC6739420; doi:10.1016/j.cub.2019.07.083)
Supplement: Document S1. Figures S1–S7 [file mmc1.pdf]

Current Biology, Volume *29*

Supplemental Information

Genetically Defined Functional Modules for Spatial Orienting in the Mouse Superior Colliculus

Laura Masullo, Letizia Mariotti, Nicolas Alexandre, Paula Freire-Pritchett, Jerome Boulanger, and Marco Tripodi

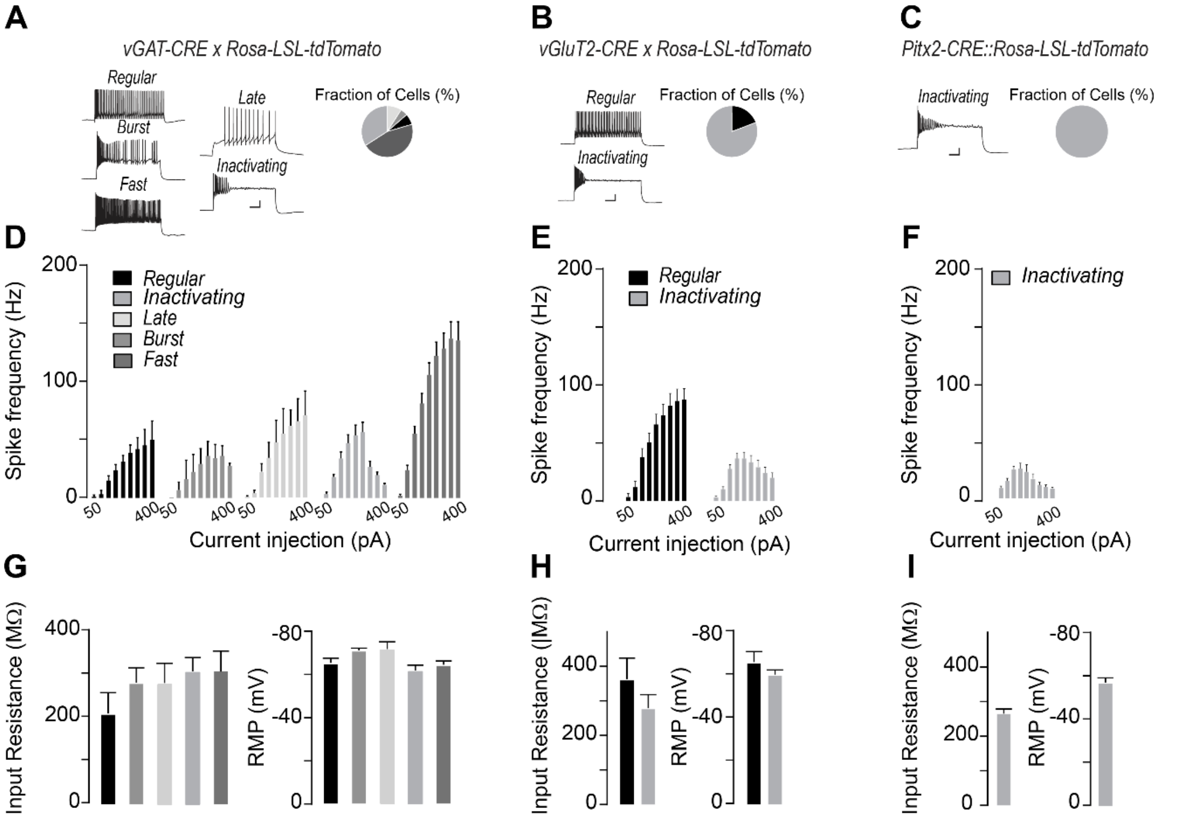

Figure S1 │ *In vitro* characterization of vGAT^{ON}, vGluT2^{ON}, and Pitx2^{ON} neurons in the SC motor domain. Related to Figure 1.

A-C, Representative firing profiles of vGAT^{ON} (**A**), vGluT2^{ON} (**B**), and Pitx2^{ON} (**C**) neurons (scale bars: 20 mV, 100 ms; 200 pA of current injected). Inset: fraction of cells per firing profile. **D-F**, Membrane potential response to steps of increasing positive current (50-400 pA) in vGAT^{ON} ($n_{NEURONS}$ = 46, n_{MICE} = 17) (**D**), vGluT2^{ON} ($n_{NEURONS}$ = 26, n_{MICE} = 5) (**E**), and Pitx2^{ON} (n_{NEURONS} = 16, n_{MICE} = 5) (**F**) neurons clustered according to their firing profile. **G-I**, Input resistance and resting membrane potential (RMP) of vGAT^{ON} (**G**), vGlut2^{ON} (**H**) and Pitx2^{ON} (**I**) neurons. All results are presented as mean ± SEM.

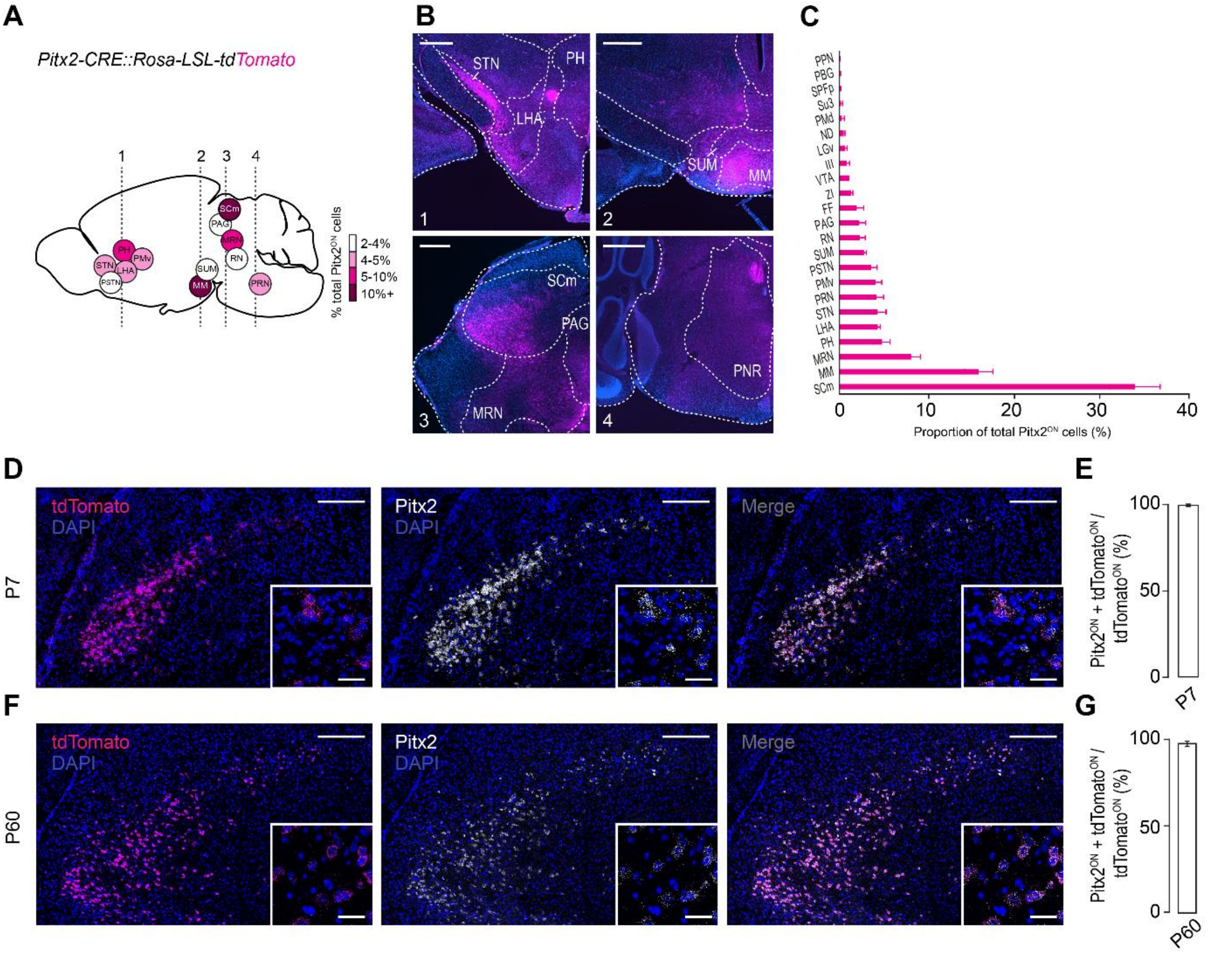

Figure S2 │ Pitx2 expression in mouse brain. Related to Figure 1.

A, Schematic representation of the top 12 brain regions containing Pitx2^{ON} cells, studied in a *Pitx2-CRE::Rosa-LSL-tdTomato* mouse ($n_{ANIMALS}$ = 3). **B**, Representative histologies highlighting presence of Pitx2^{ON} cells in coronal sections marked with dashed lines in **A** (scale bars: 500 µm). **C**, Quantification of distribution of Pitx2^{ON} cells across the brain, studied in a *Pitx2-CRE::Rosa-LSL-tdTomato* mouse ($n_{ANIMALS}$ = 3). **D-G**, Representative histologies and quantification of double *in situ* hybridization in *Pitx2Cre::Rosa-LSL-tdTomato* mice at P7 (**D, E**) and P60 (**F, G**), with *tdTomato* and *Pitx2* probes (scale bar: 100 µm, insets: 30 µm; n_{SLICES} = 6). All results are presented as mean ± SEM.

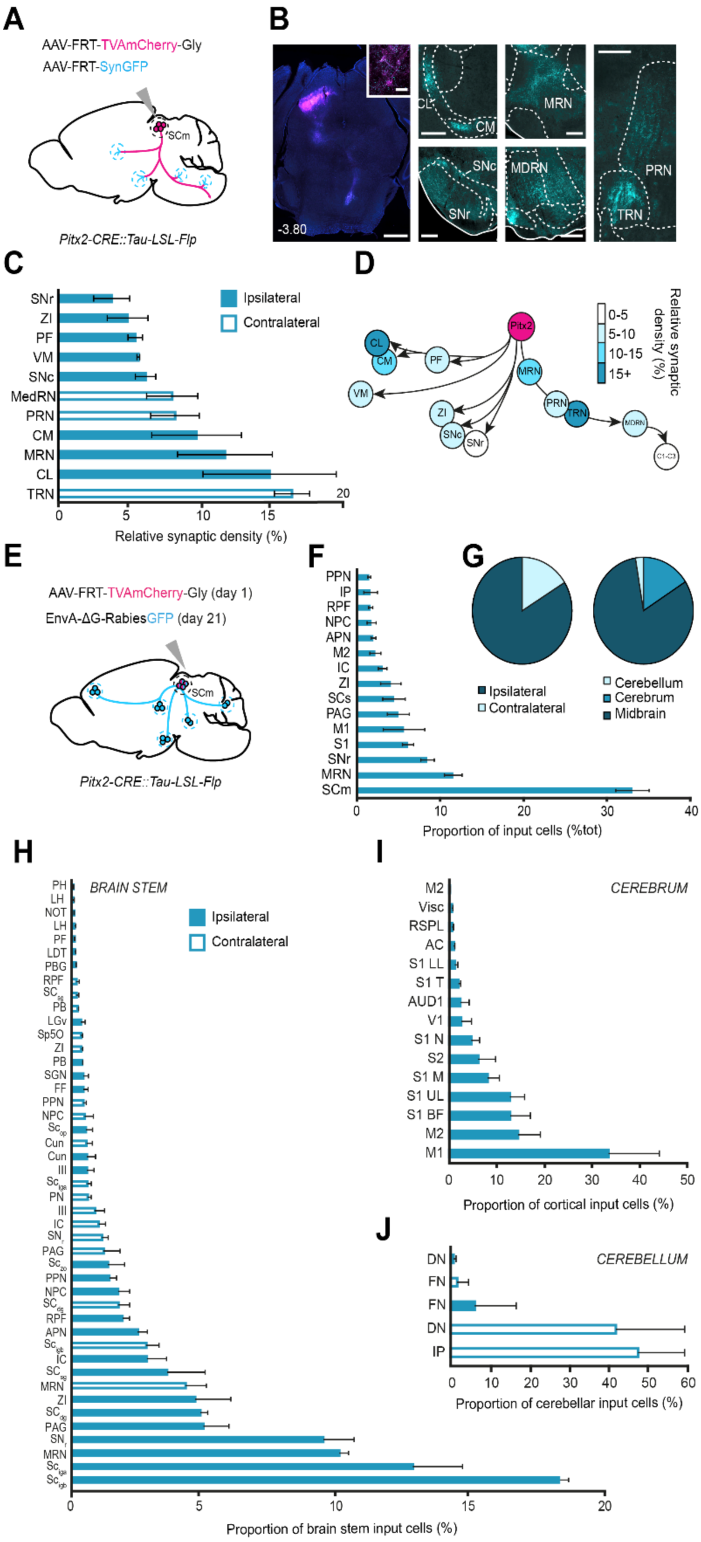
A
AAV-FRT-TVAmCherry-Gly
AAV-FRT-SynGFP
SCm
Pitx2-CRE::Tau-LSL-Flp
B
-3.80
CL
CM
MRN
SNc
SNr
MDRN
PRN
TRN
C
SNr
ZI
PF
VM
SNc
MedRN
PRN
CM
MRN
CL
TRN
Ipsilateral
Contralateral
0
5
10
15
20
Relative synaptic density (%)
D
Pitx2
CL
CM
PF
VM
MRN
PRN
TRN
MDRN
ZI
SNc
SNr
0-5
5-10
10-15
15+
Relative synaptic density (%)
E
AAV-FRT-TVAmCherry-Gly (day 1)
EnvA-ΔG-RabiesGFP (day 21)
SCm
Pitx2-CRE::Tau-LSL-Flp
F
PPN
IP
RPF
NPC
APN
M2
IC
ZI
SCs
PAG
M1
S1
SNr
MRN
SCm
0
10
20
30
40
Proportion of input cells (%tot)
G
Ipsilateral
Contralateral
Cerebellum
Cerebrum
Midbrain
H
BRAIN STEM
Ipsilateral
Contralateral
Proportion of brain stem input cells (%)
I
CEREBRUM
M2
Visc
RSPL
AC
S1 LL
S1 T
AUD1
V1
S1 N
S2
S1 M
S1 UL
S1 BF
M2
M1
0
10
20
30
40
50
Proportion of cortical input cells (%)
J
CEREBELLUM
DN
FN
FN
DN
IP
0
10
20
30
40
50
60
Proportion of cerebellar input cells (%)

Figure S3 │ Viral tracing of pre- and post-synaptic networks of the Pitx2^{ON} population. Related to Figures 2 and 7.

A, Anterograde tracing of Pitx2^{ON} neurons projections in *Pitx2-CRE::Tau-LSL-FLPo-INLA* mice targeted via FLP-dependent AAVs expressing synaptic (*cyan*, SynGFP) and membrane (*magenta*, TVAmCherry) markers (n_{MICE} = 3). **B**, Overview of infected neurons (scale bar: 1 mm, inset: 50 µm) and their extra-collicular targets (scale bars: 300 µm). **C-D**, Schematic representation (**C**) and quantification (**D**) of relative synaptic density of Pitx2^{ON} neurons terminals in target areas (Central lateral nucleus of the thalamus, CL; Central medial nucleus of the thalamus, CM; Ventral medial nucleus of the thalamus, VM; Parafascicular nucleus, PF; Zona incerta, ZI; Substantia nigra pars compacta, SNc; Substantia nigra pars reticulata, SNr; Midbrain reticular nucleus, MRN; Pontine reticular nucleus, PRN; Tegmental reticular nucleus, TRN; Medullary reticular nucleus, MDRN; Spinal cord, cervical segments C1-C3; n_{MICE} = 3). **E**, **F**, Schematic representation (**E**) and quantification (**F**) of top 15 input regions to Pitx2^{ON} neurons, studied in *Pitx2-CRE::Tau-LSL-FLPo-INLA* mice via FLP-dependent AAV expressing TVA-Gly (*magenta*, TVAmCherry) followed by EnvA-ΔG-Rabies^{GFP} (*cyan*, GFP) injection (n_{MICE} = 3). **G**, Distribution of pre-synaptic partners of Pitx2^{ON} neurons in ipsi- and contralateral hemispheres to injection site (left) and across brain divisions (right). **H-J**, Distribution of input cells in cerebrum (**H**), cerebellum (**I**) and brain stem (**J**), expressed as percentage of total input cells in relative brain division (n_{MICE} = 3). All results are presented as mean ± SEM.

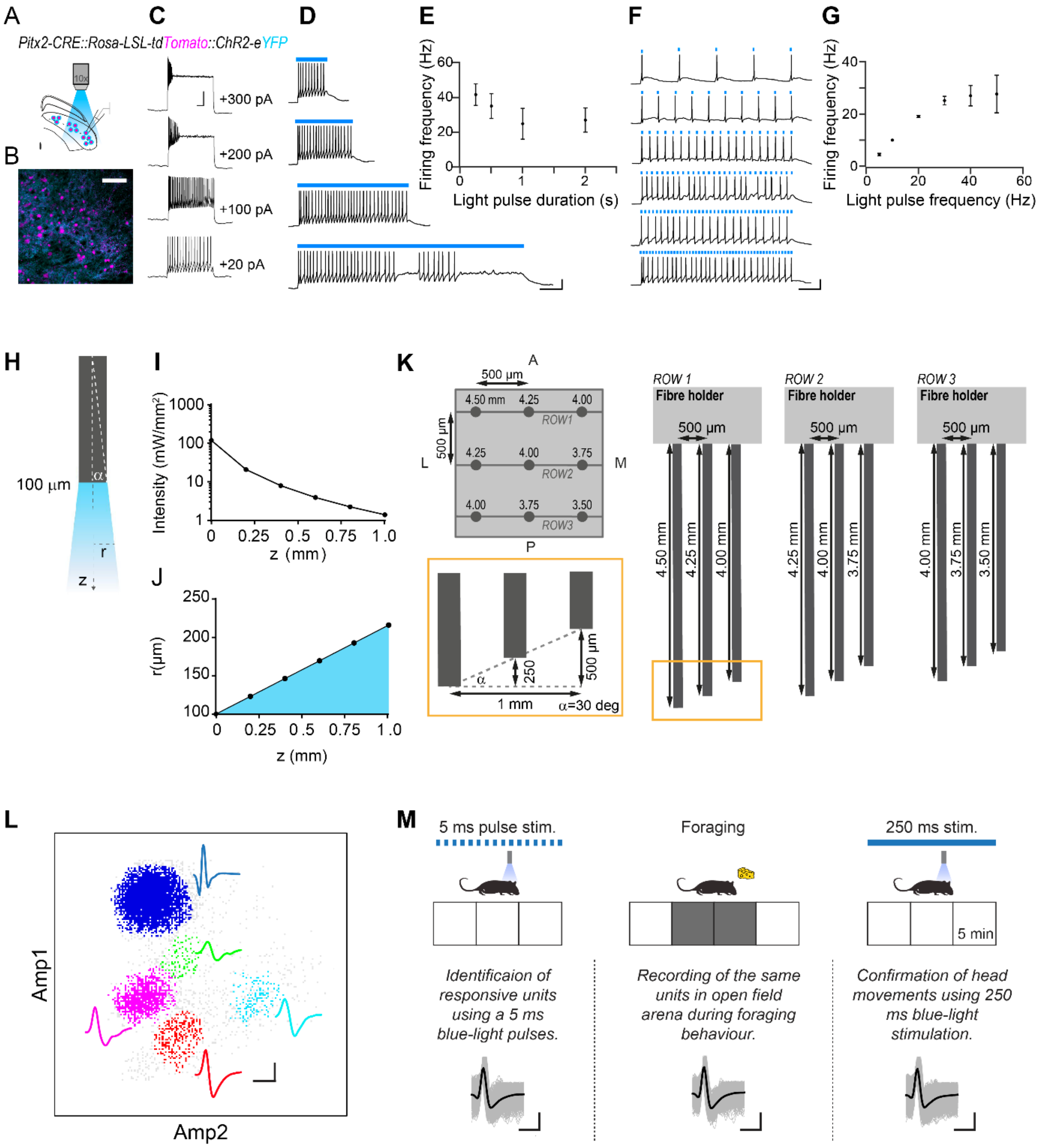

Figure S4 │ *In vitro* responses of ChR2 expressing Pitx2^{ON} neurons to various regimes of optical stimulation, specifics for single optic fibre and optic fibre array, and optotagging paradigm. Related to Figures 2, 4 and 6.

A, **B**, Schematic of recording setup (**A**) and visual targeting of Pitx2^{ON} neurons in slices from *Pitx2-CRE::Rosa-LSL-tdTomato::ChR2-eYFP* mice (**B**) (scale bar: 100 µm). **C**, Membrane potential response to steps of increasing positive current (20-300 pA current injected; scale bars: 100 ms, 20 pA). **D**, **E**, Membrane potential (**D**) and firing frequency (**E**) in response to blue light pulses of increasing duration. **F**, **G**, Membrane potential (**F**) and firing frequency (**G**) in response to 5 ms blue light pulses of increasing frequency (5-50 Hz) (scale bar: 20 ms, 20 pA; $n_{NEURONS}$ = 7, n_{MICE} = 2). **H**, Schematic of light diffusion from optic fibre used for optogenetic stimulation *in vivo*. **I**, **J**,

Calculations of variation in light intensity (**I**) and radius (r) of light cone (**J**) as a function of the distance from fibre tip (z). **K**, Specifications of custom-made 3x3 optic fibre array and implant orientation with respect to mouse head; fibres are spaced at a distance of 500 µm along both the mediolateral and anteroposterior axes and fibre length is designed to accommodate for the spherical surface of the motor SC. All results are presented as mean ± SEM. **L**, Action potential amplitudes on two channels of a tetrode bundle showing five amplitude clusters (colour coded). Average action potential waveforms from the maximum amplitude channel are shown next to each cluster (scale bars: 50 µV, 2 ms). **M**, Experimental design: three epochs of 5 minutes each were recorded while the animal was free to explore an open field arena; in these epochs, 5 ms pulse stimulation at 30 Hz was used to isolate the responsive units. The same units were recorded during the following four epochs (two in light conditions, white blocks; and two in dark conditions, grey blocks) where the animal was exposed to soy milk drops. The last recording session consisted of three epochs during which 250 ms of continuous blue light stimulation was applied in order to confirm the characteristic vector for each animal recorded. Insets show the consistency of spike waveforms from a representative responsive unit across the three different recording sessions. Average action potential waveforms is in black, single action potential are in grey (scale bars: 50 µV, 2 ms).

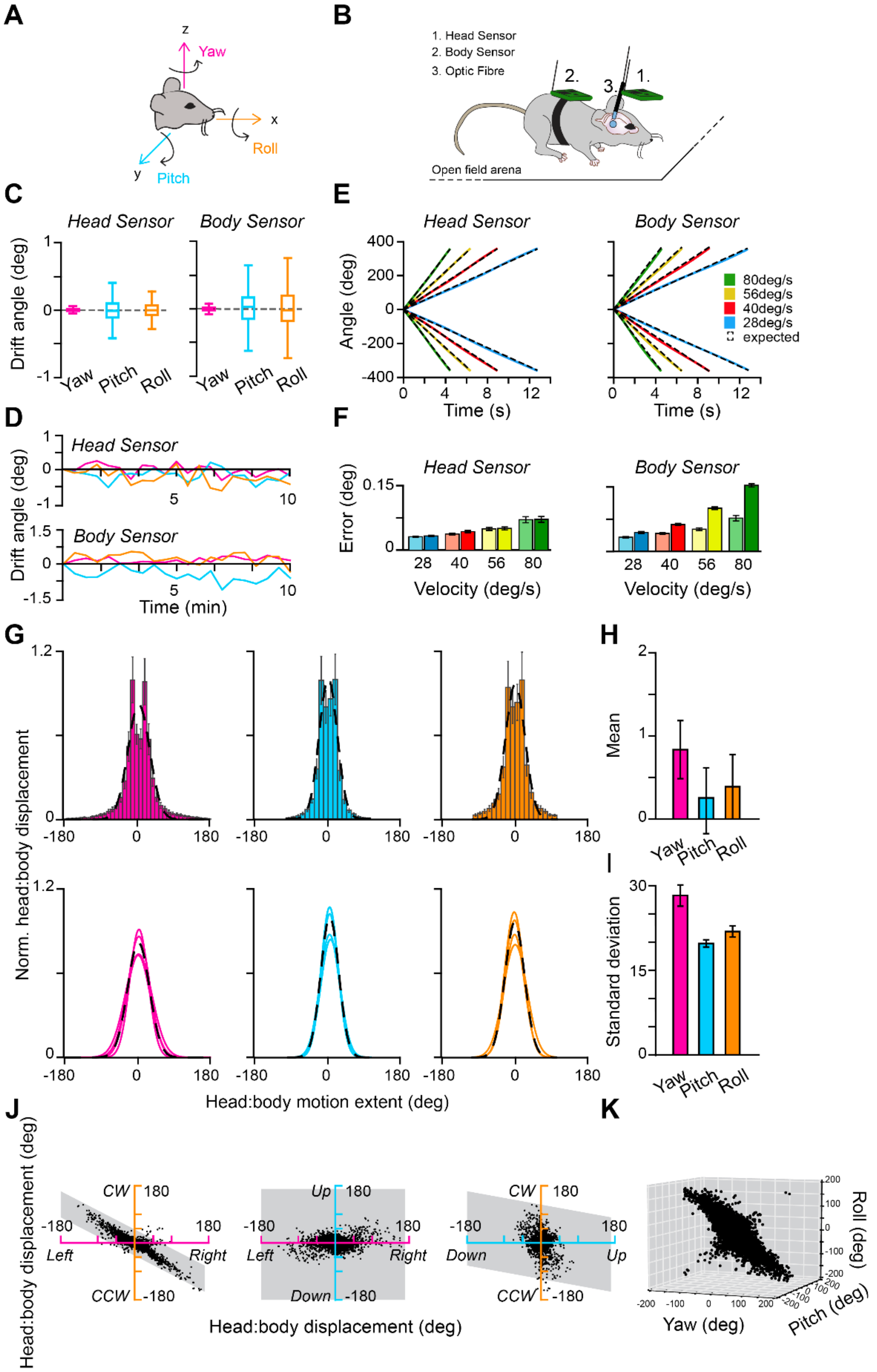
A
B
1. Head Sensor
2. Body Sensor
3. Optic Fibre
Open field arena
Yaw
Pitch
Roll
C
Head Sensor
Body Sensor
Drift angle (deg)
D
Time (min)
E
Angle (deg)
Time (s)
80deg/s
56deg/s
40deg/s
28deg/s
expected
F
Error (deg)
Velocity (deg/s)
G
Norm. head:body displacement
Head:body motion extent (deg)
H
Mean
I
Standard deviation
J
Head:body displacement (deg)
Left
Right
Up
Down
CW
CCW
K
Yaw (deg)
Pitch (deg)
Roll (deg)

Figure S5 │ Stability of double sensor readings and movement kinematics. Related to Figure 2.

A, Schematic of the colour-coded axes of rotation: yaw (*magenta*), pitch (*blue*) and roll (*orange*). **B**, Schematic of the *in vivo* optical stimulation recording setup with optic fibre and two sensor boards. **C**, Box and whisker plots showing the jitter in the sensors during static regime for each Eulerian dimension. **D**, Cumulative drift of the two sensors over 10 minute recordings in static regime. **E**, Sensors output recorded during step-motor-driven rotations over 360° at four different speeds and two directions (coloured lines) plotted against the expected trajectory (black dashed line). **F**, Bar plots showing the error for each sample at each speed (light shades for clockwise rotations and dark shades for counter-clockwise rotations; $p = 0.08$). **G**, Average head-over-body displacement for each axis of rotation recorded from freely moving animals, normalised to the maximum displacement produced ($n_{\mathrm{MICE}} = 4$) (top). Single animal average (solid lines) and population average (dashed line) of the Gaussian curve fitted to the histogram of head-over-body displacements ($n_{\mathrm{MICE}} = 4$) (bottom). **H**, **I**, Mean standard error (**H**) and mean standard deviation (**I**) of the Gaussian curves fitted in **G**. **J, K**, Eulerian representation of conjunctive motion from one representative mouse (**J**) and three-dimensional representation of yaw, pitch and roll displacements for all the mice (**K**) ($n_{\mathrm{MICE}} = 4$). All results are presented as mean ± SEM.

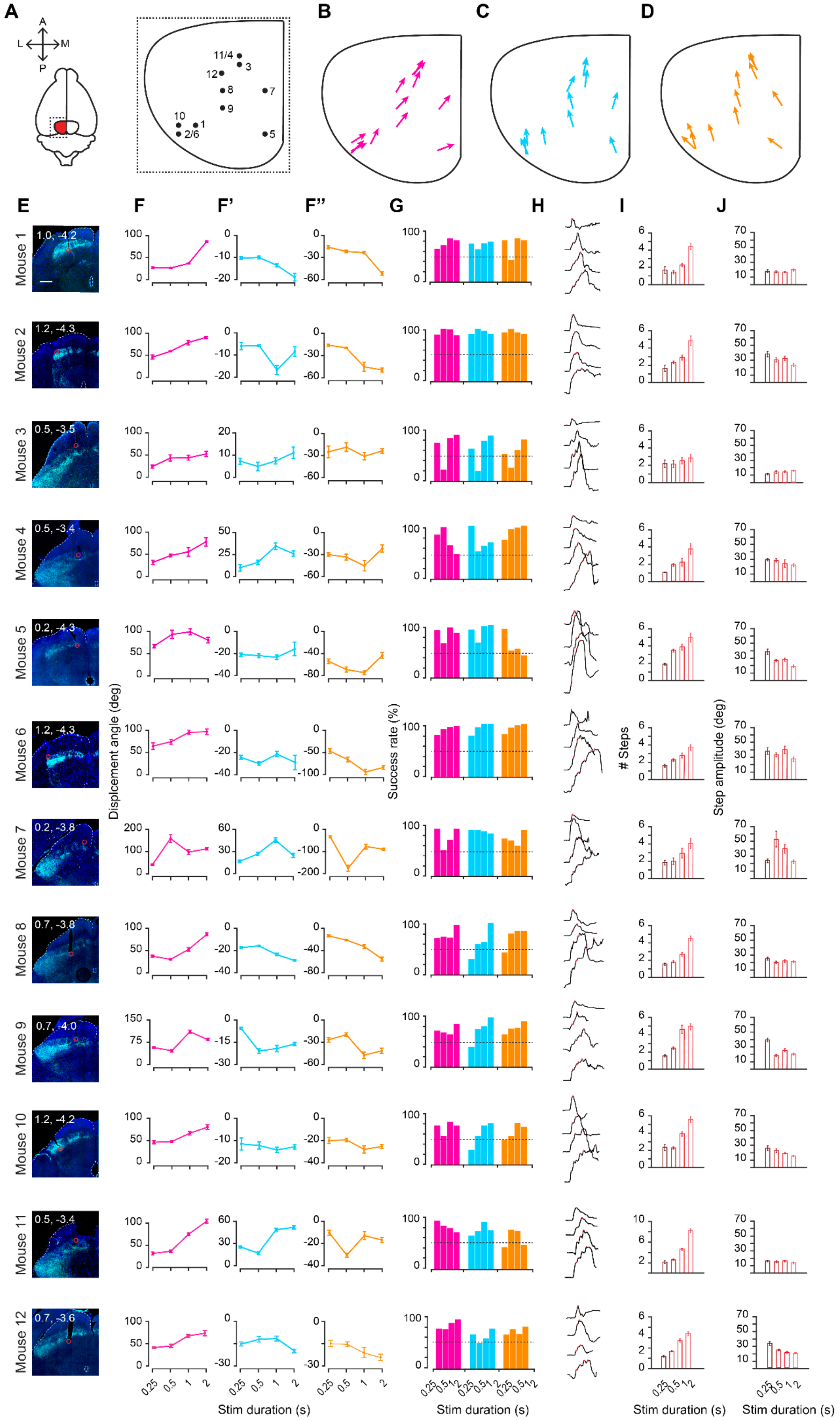
A
B
C
D
E
F
F'
F''
G
H
I
J
A
L
M
P
11/4
3
12
8
7
9
10
1
2/6
5
Mouse 1
Mouse 2
Mouse 3
Mouse 4
Mouse 5
Mouse 6
Mouse 7
Mouse 8
Mouse 9
Mouse 10
Mouse 11
Mouse 12
1.0, -4.2
1.2, -4.3
0.5, -3.5
0.5, -3.4
0.2, -4.3
1.2, -4.3
0.2, -3.8
0.7, -3.8
0.7, -4.0
1.2, -4.2
0.5, -3.4
0.7, -3.6
Displacement angle (deg)
Success rate (%)
Steps
Step amplitude (deg)
Stim duration (s)
Stim duration (s)
Stim duration (s)
Stim duration (s)

Figure S6 │ Reliability of head kinematics driven by optical stimulation of Pitx2 neurons. Related to Figures 2 and 3.

A-D, Top view summary of implant locations (**A**) and summary of characteristic movement vector produced in yaw (**B**), pitch (**C**) and roll (**D**) for animals shown in **E-J**. **E**, Histology of the implantation site for each tested mouse with estimated implant coordinates (x, y; scale bar: 500 µm)**.** **F-H**, Effect of stimulus duration on the head displacement angle (**F-F"**) and the success rate of a head movement bout (**G**) for each mouse for yaw (*magenta*), pitch (*blue*) and roll (*orange*). Kinematics of head displacements for each mouse along the yaw dimension for increasing duration of stimulation (0.25, 0.5, 1 and 2 s); note the presence of stepwise trajectories for prolonged stimulations (**H**). **I-J**, Trial-by trial average of the number of recorded steps (**I**) and average step amplitude (**J**) for each mouse for increasing stimulus duration (n_{MICE} = 12, $n_{TRIALS/MOUSE}$ = 60). All results are presented as mean ± SEM.

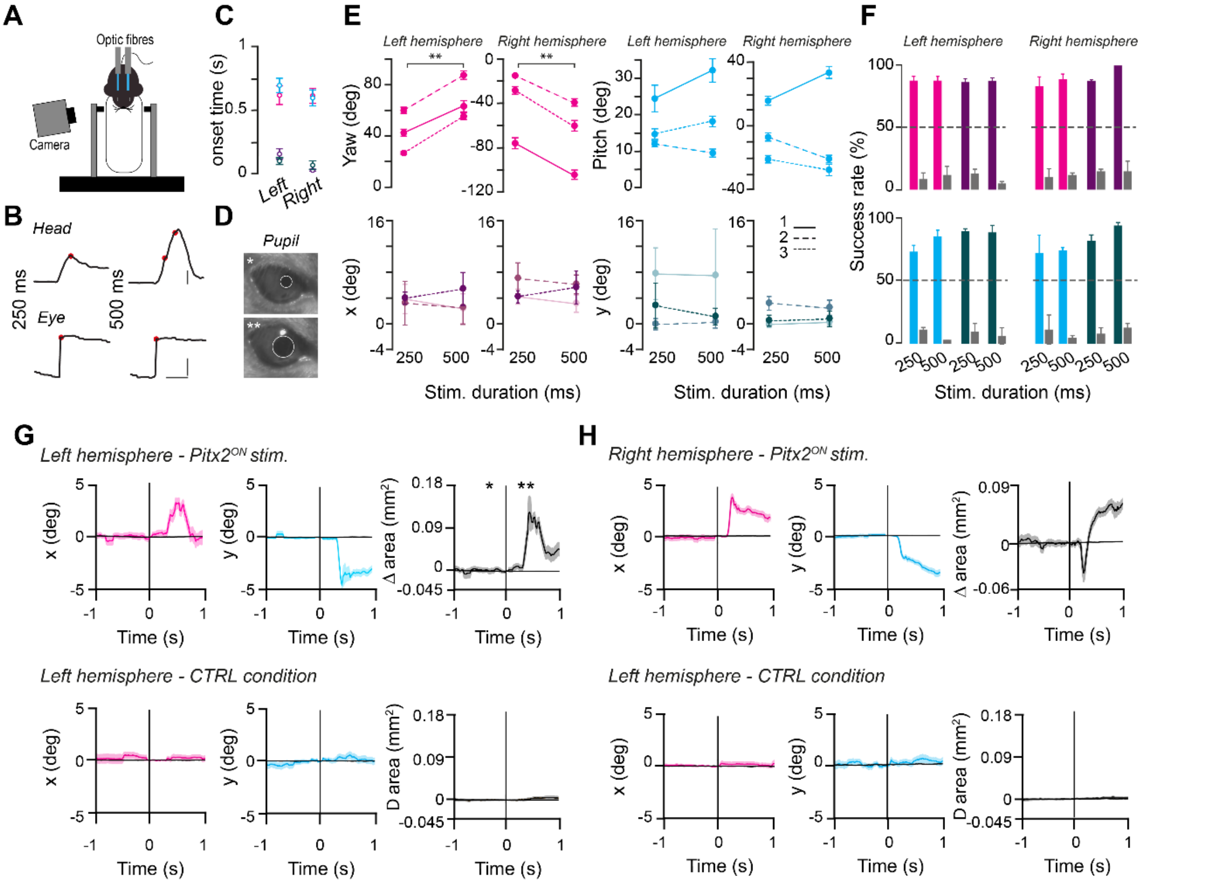

Figure S7 │ Eye and head movements elicited by optogenetic stimulations. Related to Figure 2.

A, Schematic of experimental setup for eye tracking in head restrained *Pitx2-CRE::Rosa-LSL-ChR2-eYFP* animals. Two optic fibres were implanted: one on the left and one on the right hemisphere to record movements evoked by a contra- or ipsi-lateral SC stimulation with respect to the monitored eye (right eye). **B**, Representative traces for yaw component for the head movement and x component for the eye movement after 250 (left) or 500 ms (right) of blue light stimulation of the left hemisphere. Red dots mark a change in velocity for the head and the eye motion (for head displacement, scale bars: 20 deg, 1 s; for eye movement, scale bars: 2 deg, 1 s). **C**, Time of onset of motion after blue light stimulation for yaw (*magenta*) and pitch (*blue*) and for x (*purple*) and y (*dark blue*) ($n_{MOUSE} = 3$; $n_{TRIALS/MOUSE} = 30$). **D**, Frames from the eye camera showing changes in pupil dilation (see panel **G**) taken before (*one asterisk*) and after (*two asterisks*) 250 ms blue light stimulation with fitted ellipses (*white*). **E**, Effect of stimulus duration on the head displacement angle for yaw and pitch (top), and on the eye movement for x and y after the stimulation of the left or right hemisphere ($n_{MOUSE} = 3$; $n_{TRIALS/MOUSE} = 30$; $p_{LEFT,YAW} = 0.008$, $p_{RIGHT,YAW} = 0.006$, $p_{LEFT,PITCH} = 0.9334$, $p_{RIGHT,PITCH} = 0.4251$, $p_{LEFT,X} = 0.11$, $p_{RIGHT,X} = 0.0712$, $p_{LEFT,Y} = 0.4367$, $p_{RIGHT,Y} = 0.72$). **F**, Effect of stimulus duration on the probability of executing a head movement bout in yaw and pitch, and eye movement in x and y after the stimulation of the left or right hemisphere. Grey bars for the effect of the stimulation in control mice in absence of ChR2 expression ($n_{MICE} = 3$, $n_{TRIALS/MOUSE} = 30$). **G**, Examples of light-triggered average (LTA) of eye movements along the x and y components, and of the pupil area (*grey*) calculated for blue light stimulation of 250 ms in the left hemisphere of the same animal in **B** and **D** expressing ChR2 in Pitx2^{ON} neurons (top) and in a control animal (bottom) ($n_{Pitx2\text{-}ChR2\ MICE} = 3$, $n_{CTRL\ MICE} = 3$, $n_{TRIALS/MOUSE} = 30$). **H**, Same as in **G** for the right hemisphere. All results are presented as mean ± SEM.
