# Supplementary material for: Genetically Defined Functional Modules for Spatial Orienting in the Mouse Superior Colliculus
Source: Curr Biol. 2019 Sep 9;29(17):2892–2904.e8. doi: 10.1016/j.cub.2019.07.083 (PMC6739420; doi:10.1016/j.cub.2019.07.083)
Supplement: Document S2. Article plus Supplemental Information [file mmc5.pdf]

Article

Current Biology

Genetically Defined Functional Modules for Spatial Orienting in the Mouse Superior Colliculus

Graphical Abstract

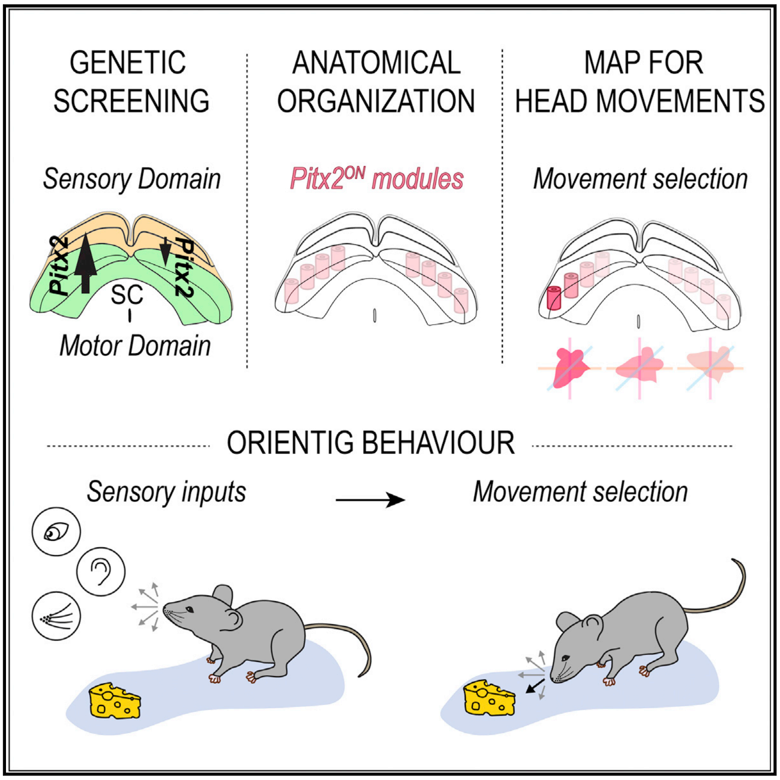

Authors

Laura Masullo, Letizia Mariotti, Nicolas Alexandre, Paula Freire-Pritchett, Jerome Boulanger, Marco Tripodi

Correspondence

mtripodi@mrc-lmb.cam.ac.uk

In Brief

Masullo and Mariotti et al. uncover the existence of genetically defined neurons in the superior colliculus responsible for the execution of three-dimensional reorienting movements. These neurons are clustered in anatomically defined modules, giving origin to a discretely organized and selectively addressable spatial-motor indexing system.

Highlights

- *Pitx2* expression labels a functionally homogeneous class of projecting SC neurons
- Pitx2^{ON} neurons drive three-dimensional head movements during foraging behavior
- Pitx2^{ON} neurons are organized in an orderly array of anatomical modules
- Modularity of Pitx2^{ON} neurons defines a discrete motor map for spatial orienting

Masullo et al., 2019, Current Biology *29*, 2892–2904
September 9, 2019 © 2019 MRC Laboratory of Molecular Biology.
Published by Elsevier Ltd.
https://doi.org/10.1016/j.cub.2019.07.083

Current Biology
Article

Genetically Defined Functional Modules for Spatial Orienting in the Mouse Superior Colliculus

Laura Masullo,[1,2] Letizia Mariotti,[1,2] Nicolas Alexandre,[1] Paula Freire-Pritchett,[1] Jerome Boulanger,[1] and Marco Tripodi[1,3,*]
[1]MRC Laboratory of Molecular Biology, Neurobiology Division, Francis Crick Avenue, Cambridge CB2 0QH, UK
[2]These authors contributed equally
[3]Lead Contact
*Correspondence: mtripodi@mrc-lmb.cam.ac.uk
https://doi.org/10.1016/j.cub.2019.07.083

SUMMARY

In order to explore and interact with their surroundings, animals need to orient toward specific positions in space. Throughout the animal kingdom, head movements represent a primary form of orienting behavior. The superior colliculus (SC) is a fundamental structure for the generation of orienting responses, but how genetically distinct groups of collicular neurons contribute to these spatially tuned behaviors remains largely to be defined. Here, through the genetic dissection of the murine SC, we identify a functionally and genetically homogeneous subclass of glutamatergic neurons defined by the expression of the paired-like homeodomain transcription factor Pitx2. We show that the optogenetic stimulation of Pitx2^{ON} neurons drives three-dimensional head displacements characterized by stepwise, saccade-like kinematics. Furthermore, during naturalistic foraging behavior, the activity of Pitx2^{ON} neurons precedes and predicts the onset of spatially tuned head movements. Intriguingly, we reveal that Pitx2^{ON} neurons are clustered in an orderly array of anatomical modules that tile the entire intermediate layer of the SC. Such a modular organization gives origin to a discrete and discontinuous representation of the motor space, with each Pitx2^{ON} module subtending a defined portion of the animal's egocentric space. The modularity of Pitx2^{ON} neurons provides an anatomical substrate for the convergence of spatially coherent sensory and motor signals of cortical and subcortical origins, thereby promoting the recruitment of appropriate movement vectors. Overall, these data support the view of the superior colliculus as a selectively addressable and modularly organized spatial-motor register.

INTRODUCTION

In order to successfully interact with the environment, animals need to produce orienting movements toward specific positions in space. Whether it is to hit a tennis ball or to shift our gaze toward an approaching threat, we are in constant need of planning and performing spatially accurate motor plans to match our goals. This relies on the ability to reconstruct the location of objects of interest in space by integrating sensory information of distinct modalities; this information is then used to guide the selection and execution of appropriate actions.

At the neuronal level, this process requires the convergence of target-related and internally generated signals onto a motor output network able to produce appropriate movement vectors. Meaningful integration of this information is what creates the relational map that transforms object location, as probed by the senses, in motor coordinates, defining our ability to interact with the world around us and giving origin to our sense of space [1, 2].

The mammalian superior colliculus (SC) is a prominent site of convergence of sensory information of multiple modalities, which is integrated locally and employed to trigger appropriate motor responses [3–5]. The lamination of this midbrain region defines its organization into functional domains. Superficial layers are defined as the visual sensory domain of the SC, receiving retinotopically organized information directly from the eye and indirectly from visual and extrastriate cortical regions [6]. The deeper layers of the SC form its multimodal motor domain, where somatosensory and auditory information is conveyed together with motor and internal state-related inputs [3, 7–9]. A proportion of neurons in these layers display motor-related activity, being directly involved in the initiation of spatially accurate motor programs [10–12].

The repertoire of SC-mediated motor functions is extremely diverse, both within and between species. The SC has been implicated in the generation of a vast range of orienting behaviors, primarily head and eye movement, but also rotations of the pinnae and displacement of whiskers [10, 12–16], particularly evident in animals that extensively employ auditory cues and somatosensation to locate objects in space. To further add to the complexity of SC-mediated motor behaviors, this midbrain region has also been implicated in the generations of sensory-driven appetitive and defensive innate behaviors [17, 18], such as escaping and freezing [19].

While these functional observations have been instrumental in defining a role for the SC as a universal encoder of spatially tuned behaviors, it remains largely unknown how functionally distinct neuronal groups within the SC are organized to support the production of such diverse motor outputs, an issue made more

 © 2019 MRC Laboratory of Molecular Biology. Published by Elsevier Ltd.
This is an open access article under the CC BY license (http://creativecommons.org/licenses/by/4.0/).

pressing by the prominent functional heterogeneity that has been observed across neurons in the motor domain of the SC [20, 21]. The inability to study functional collicular populations in isolation hinders our potential to fully characterize principles of circuit organization and strategies for sensorimotor transformation implemented locally. One of the reasons why the intrinsic circuit organization of the SC remains elusive is the lack of genetic characterization of the neuronal populations of the motor SC. To overcome this limitation, it would be valuable to translate the early line of physiological studies from primates and cats into a genetically tractable model, such as the mouse. While only a few studies have, to date, investigated the role of the SC in the control of head movements in rodents [17, 22–24], the recent identification of collicular neurons involved in the production of head movement vectors in mice [23] makes this transition possible.

Here, taking advantage of the genetic toolkit of the mouse, we combine a gene expression screen with the functional characterization of neuronal diversity within the motor domain of the SC. By doing so, we identify a subpopulation of glutamatergic projecting neurons located in the *stratum griseum intermediale* (SGI) and defined by the expression of the paired-like homeodomain transcription factor Pitx2 [25], accounting for approximately half of the local glutamatergic population. We show that individual Pitx2^{ON} units are tuned to specific head displacement vectors and that their optogenetic activation is sufficient to trigger stereotyped head orienting movements characterized by stepwise kinematics. Strikingly, Pitx2^{ON} neurons cluster in anatomically segregated modules that are the direct target of known patchy subcortical afferents. Our results suggest that the modular distribution of Pitx2^{ON} neurons defines an orderly and discrete array of functional modules for spatial orienting within the SC, with each module attending a specific portion of space. We propose that the uncovered modularity serves a spatial logic by providing a site of convergence for coherent sensory and motor signals of cortical and subcortical origin, which is in turn instrumental for the selection and execution of appropriate spatial orienting movements. These findings provide experimental support to the early proposition of spatial-motor coherency of SGI afferent patches [26–28] and point to the role of the SC as a modularly organized and selectively addressable spatial-motor register.

RESULTS

Pitx2 Expression Defines a Functionally Homogeneous Glutamatergic Subpopulation in the SGI

The first step toward a genetic dissection of collicular motor circuits is the assessment of the degree of functional heterogeneity that exists among SGI neurons. Previous studies have highlighted the existence of a considerable degree of electrophysiological diversity among SGI neurons [20, 21] and suggested the existence of independent functional channels within the SC [18, 29]. However, the lack of information about the molecular underpinnings of the observed functional heterogeneity has limited our ability to selectively target functionally defined collicular neurons.

We recorded the electrophysiological properties of SGI neurons in acute slices from wild-type (WT) mice and used a hierarchical clustering algorithm to study the extracted neuronal features [30] in order to obtain an unbiased and quantitative picture of the intrinsic functional diversity of SGI neurons. This analysis, in line with earlier work in rats [21], revealed the existence of five functional classes of SGI neurons (Figures 1A and 1B).

While far from being exhaustive, such an unbiased functional classification offers the opportunity to assess the degree to which genetic differences contribute to functional heterogeneity.

Given that a primary determinant of functional diversity is likely to relate to the excitatory or inhibitory nature of the recorded neurons, we crossed *Rosa-LoxP-STOP-LoxP (LSL)-tdTomato* [31] reporter mice to either *vGluT2-CRE* or *vGAT-CRE* mice [32] and selectively targeted excitatory or inhibitory neurons, respectively, for recordings (Figures 1B and 1C; Figures S1A, S1B, S1D, S1E, S1G, and S1H). This revealed that vGluT2^{ON} neurons predominantly populated cluster I and V of the similarity matrix, whereas vGAT^{ON} neurons spread broadly throughout all classes (Figure 1C).

These data indicate that, while excitatory and inhibitory neurons together account for all functional neuronal types characterized in WT animals, neither of them uniquely identifies a single functional class, highlighting the need for further refinement in characterizing the genetic diversity within the SGI. To address this, we screened gene expression in the SC by extracting RNA from sensory and motor layers of the SC at post-natal day (P)7 (Figure 1D) and sequenced the derived cDNA libraries. We analyzed differential gene expression between the two functional domains of the SC with the aim of identifying transcripts virtually exclusive to the motor layers, thus representing a convenient experimental tool for targeting *in vivo*.

The analysis of differential gene expression between the two layers identified RNAs enriched in the motor domain (Figures 1E and 1F; Table S1). In particular, the mRNA encoding for the paired-like homeodomain transcription factor Pitx2 [25] was selectively enriched in the SGI and virtually absent from more superficial layers of the SC (Figures 1E and 1F; Log2FoldChange = 2.79; p = 7.477E-04), thereby providing the first candidate molecular marker to isolate a genetically distinct population of neurons in the SGI.

To validate our sequencing data, we crossed *Pitx2-CRE* mice [33] with a *Rosa-LSL-tdTomato* reporter line. Pitx2^{ON} neurons were present in a few forebrain areas but most prominently in the SC (Figures 1G and 1H; Figure S2). In agreement with the results of the genetic screen, Pitx2^{ON} neurons were excluded from the superficial layers of the SC and solely present in the SGI, in which they account for 23% ± 2% of all neurons. We then asked whether this genetically identified neuronal population also shares unique physiological features. First, we performed patch-clamp recordings of *Pitx2-CRE::Rosa-LSL-tdTomato* neurons in acute slice preparation (Figures 1C and 1I, Figures S1C, S1F, and S1I). We observed the same electrophysiological profile in all Pitx2^{ON} neurons recorded; indeed, the clustering algorithm grouped their electrophysiological parameters in a single cluster in the similarity matrix (cluster V; Figure 1C). With respect to the neurotransmitter identity of Pitx2^{ON} neurons, multiplex triple *in situ* hybridization using *tdTomato*, *vGluT2*, and *vGAT* probes in *Pitx2-CRE::Rosa-LSL-tdTomato* mice revealed that all Pitx2^{ON} neurons express exclusively *vGluT2* RNA (Figure 1J) and that they represent about half of all

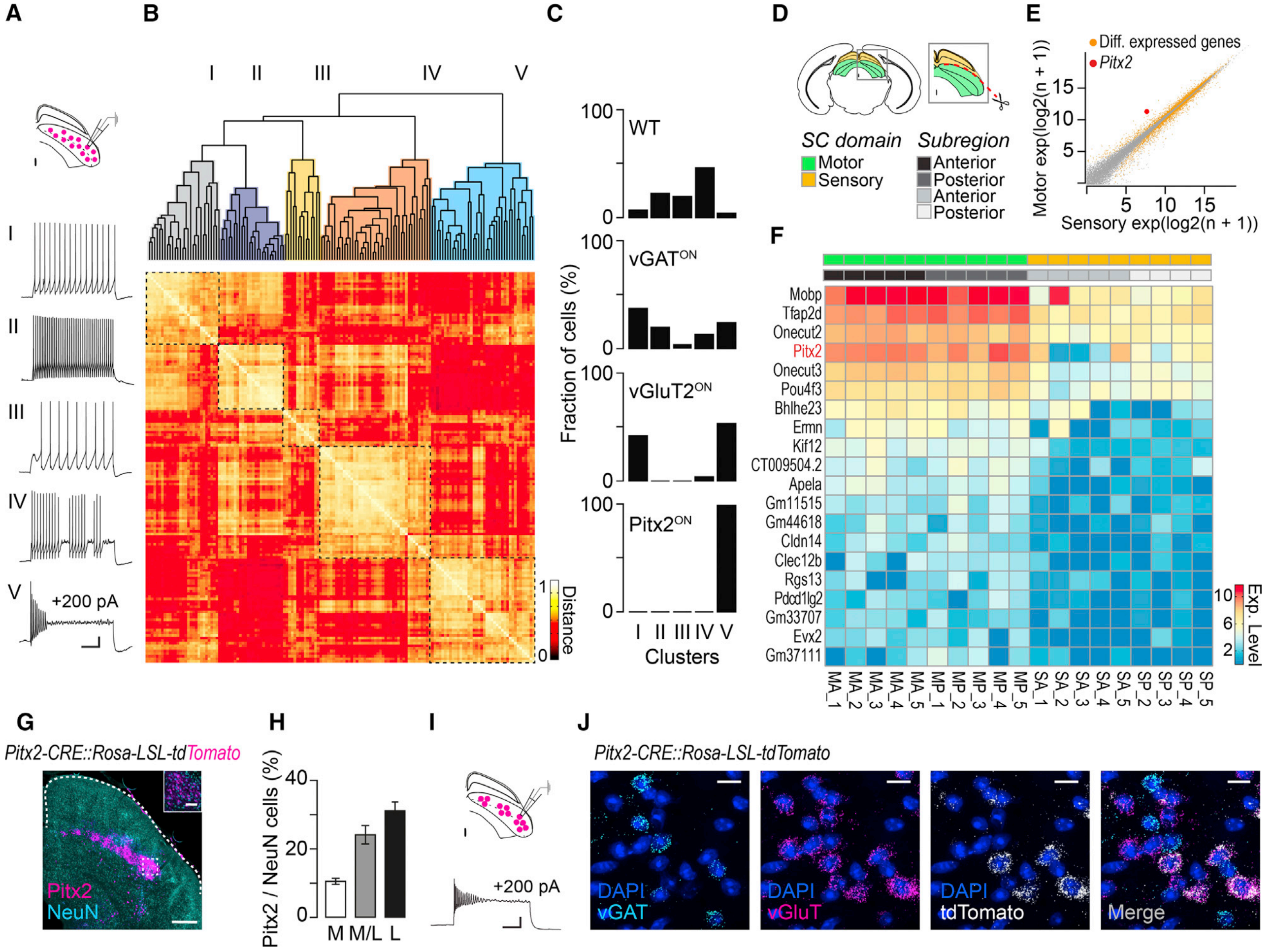

Figure 1. *Pitx2* Expression Defines a Functionally Homogeneous Glutamatergic Neuronal Subpopulation in the SGI

(A) Schematic of patch-clamp recording from collicular neurons expressing tdTomato and five representative firing profiles recorded from wild-type animals (scale bars: 20 mV, 100 ms; 200 pA current injected).

(B and C) Hierarchical clustering of SGI neurons based on electrophysiological parameters obtained from recordings of wild-type ($n_{NEURONS}$ = 64, n_{MICE} = 21), vGAT^{ON} ($n_{NEURONS}$ = 46, n_{MICE} = 17), vGluT2^{ON} ($n_{NEURONS}$ = 26, n_{MICE} = 5), and Pitx2^{ON} ($n_{NEURONS}$ = 16, n_{MICE} = 5) neurons (B) and relative distribution for each cluster (C).

(D–F) Schematic of SC dissection for RNA sequencing (D) and scatterplot of mean normalized read counts for each gene between sensory (superficial) and motor (deep) SC domains. Genes highlighted in orange are significantly differentially expressed (padj $\leq$ 0.05). *Pitx2* is highlighted in red (E). Heatmap of 20 most significantly enriched genes in the motor versus sensory domain of the SC, with levels of expression (Exp. Level; a.u.) assessed in sensory and motor SC domain fractions (MA, motor anterior; MP, motor posterior; SA, sensory anterior; SP, sensory posterior) across replicates (F) (n_{MICE} = 5). Pitx2 is highlighted in red.

(G and H) Distribution of Pitx2^{ON} neurons in the SGI of *Pitx2Cre::Rosa-LSL-tdTomato* mice (scale bars: 250 µm; inset 50 µm) (G) and their fraction across the medial-lateral extent of the SGI ($n_{NEURONS}$ = 16,184 for NeuN; 3,793 for Pitx2; n_{SLICES} = 9; M, medial; M/L, medial-lateral; L, lateral) (H).

(I) Whole-cell recordings from Pitx2^{ON} neurons highlighting homogeneous inactivating firing profile and cluster pertinence (see C; scale bar: 20 mV, 100 ms; 200 pA current injected).

(J) Triple *in situ* hybridization in *Pitx2Cre::Rosa-LSL-tdTomato* mice, with *tdTomato*, *vGAT*, and *vGluT2* probes (scale bar: 20 µm; n_{SLICES} = 12). All results are presented as mean ± SEM.

See also Figures S1 and S2 and Table S1.

excitatory SGI neurons (44% ± 3%; $n_{NEURONS}$ = 4,630 vGluT2^{ON}; 1,973 Pitx2^{ON}).

Pitx2^{ON} Neurons Drive Stereotyped Head Displacements Characterized by Stepwise Kinematics

Given the glutamatergic nature of Pitx2^{ON} neurons, we considered whether they were part of the output network of the SC [20]. In order to trace the projections of Pitx2^{ON} neurons and to account and normalize for possible fluctuations in the levels of expression of *Pitx2* over time, we first crossed *Pitx2-CRE* with *Tau-LSL-FLPo-INLA* mice [34], leading to permanent expression of FLP recombinase in Pitx2^{ON} neurons; we then co-injected two FLP-dependent AAV viruses expressing a synaptic and a membrane marker, respectively AAV(1)-CMV-FRT-TVAmCherry-2A-Gly and AAV(9)-CMV-FRT-SynGFP-WPRE, in the SGI of adult mice (Figure S3A) and quantified downstream connectivity based on SynGFP intensity [35] (Figures S3B–S3D). These experiments showed that Pitx2^{ON} neurons project along the

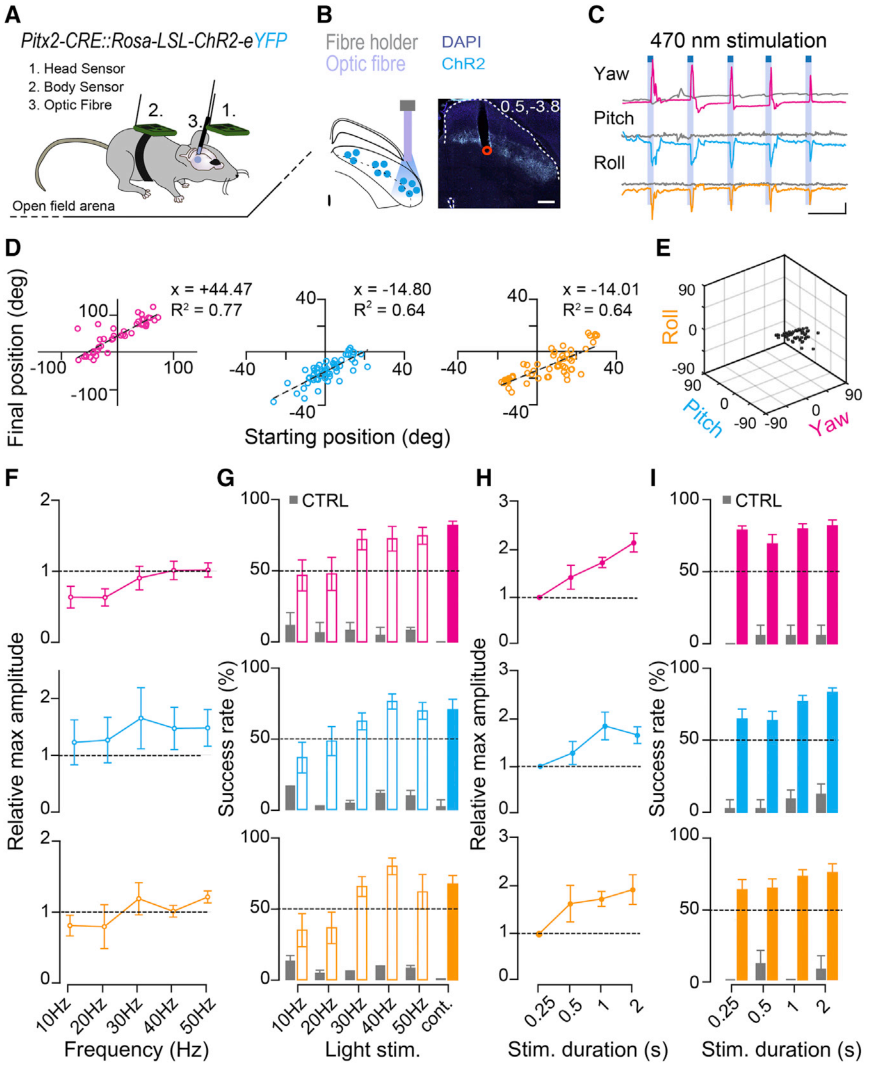

Figure 2. Pitx2^{ON} Neurons Activation Elicits Stereotypical Head Displacements in a Body-Referenced Frame

(A) Schematic representation of the optic fiber implant and sensor boards placement on head and body.

(B) Schematic of optic fiber implant and Pitx2^{ON} neurons expressing ChR2 (left). Representative SC coronal section of an implanted *Pitx2-CRE::Rosa-LSL-ChR2-eYFP* mouse with implant coordinates (x, y; scale bar: 250 μm) (right).

(C) Light-driven three-dimensional head displacements (250 ms light pulses, blue squares) in yaw (magenta), pitch (blue), and roll (orange) from a representative *Pitx2-CRE::Rosa-LSL-Chr2-eYFP*; gray traces represent head displacements along the same Eulerian axis in an implanted control animal exposed to the same optogenetic protocol in absence of ChR2 expression (scale bars: 10 s, 20 degrees).

(D) Linear correlation between starting and final head position following optogenetic stimulation for 250 ms. For yaw: positive values represent head pointing to the right relative to the midline (head and body aligned), and negative values represent head pointing to the left. For pitch: positive values represent head pointing up, and negative values represent head pointing down. For roll: positive values represents head rotated clockwise, and negative values represent head rotated counter-clockwise ($n_{TRIALS/MOUSE}$ = 60).

(E) Three-dimensional plot showing trial-by-trial reproducibility of light-driven head displacements; black spheres indicate final head-over-body position following the stimulation ($n_{TRIALS/MOUSE}$ = 60).

(F and G) Effect of stimulation frequency on the head displacement angle (F) and on the probability of executing a head movement bout for yaw (magenta), pitch (blue), and roll (orange) (G). For all frequencies tested, the stimulation duration was kept at 250 ms. For each animal, angles are normalized to the characteristic vector obtained with a 250 ms continuous (cont.) light pulse (n_{MICE} = 7, $n_{TRIALS/MOUSE}$ = 60) (G).

(H and I) Effect of stimulation duration on the head displacement angle normalized to the characteristic vector obtained with a 250 ms continuous light pulse (H) and the probability of executing a head movement bout (I) for yaw, pitch, and roll (n_{MICE} = 12, $n_{TRIALS/MOUSE}$ = 60). Gray bars represent the success rate of the stimulation in control mice in absence of ChR2 expression (n_{MICE} = 3, $n_{TRIALS/MOUSE}$ = 30) (G and I). All results are presented as mean ± SEM. Statistical values relative to (F) are p_{YAW} = 0.11; p_{PITCH} = 0.95; p_{ROLL} = 0.35. Statistical values relative to (G) are p_{YAW} = 0.08; p_{PITCH} = 0.01; p_{ROLL} = 0.01. Statistical values relative to (H) are p_{YAW} = 0.0002; p_{PITCH} = 0.03; p_{ROLL} = 0.08. Statistical values relative to (I) are p_{YAW} = 0.21; p_{PITCH} = 0.04; p_{ROLL} = 0.36. Statistically significant comparisons are, for pitch values in (G), 40 Hz versus 10 Hz (p = 0.01) and 50 Hz versus 10 Hz (p = 0.05); for roll values in (G), 40 Hz versus 10 Hz (p = 0.03) and 40 Hz versus 20 Hz (p = 0.04); for yaw values in (H), 0.25 s versus 1 s (p = 0.02), 0.25 s versus 2 s (p = 0.0001) and 0.5 s versus 2 s (p = 0.02); and for pitch values in (H), 0.25 s versus 1 s (p = 0.03).

See also Figures S3–S7 and Videos S1 and S2.

tecto-spinal tract (Figures S3B–S3D), suggesting Pitx2^{ON} neurons are a premotor population in the SC that might play an important role in the orchestration of neural activity along the cephalomotor pathway.

We then assessed whether direct activation of Pitx2^{ON} neurons could drive motor output, specifically head displacements. We therefore crossed *Pitx2-CRE* with *Rosa-LSL-ChR2-eYFP* mice [36] to drive expression of Channelrhodopsin-2 (ChR2) in Pitx2^{ON} SGI neurons and optogenetically activate them. Having validated our genetically encoded ChR2 system in slice preparation (Figures S4A–S4G), we then moved on to test for causality by optogenetic stimulation of Pitx2^{ON} neurons in freely moving mice while monitoring head-over-body kinematics using a dual inertial-sensor based approach [23] (Figure 2A; Figure S5). *Pitx2-CRE::Rosa-LSL-ChR2-eYFP* mice were implanted with a single optic fiber in the SGI (Figure 2B), and their head displacements tracked while they were free to move in an open-field arena.

Activation of Pitx2^{ON} neurons *in vivo* generated robust and precise displacements of the head relative to the body around all three main Eulerian axes of rotation (yaw, pitch, and roll;

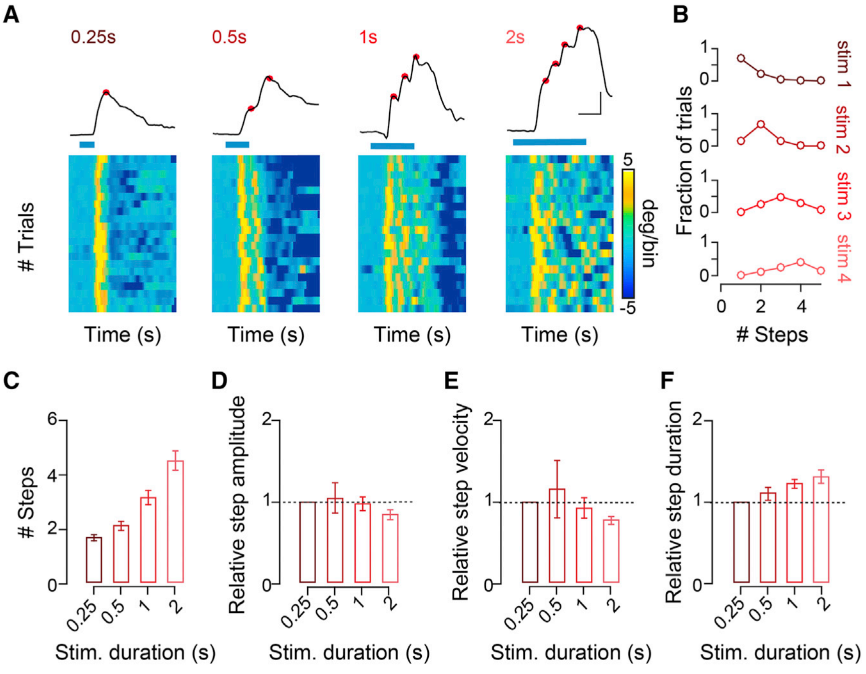

Figure 3. Pitx2^{ON} Neurons-Dependent Head Movements Are Characterized by Stepwise Kinematics

(A) Kinematics of head displacements in the yaw dimension for increasing duration of blue-light stimulation (0.25, 0.5, 1, and 2 s); note representative stepwise trajectories and deflection points (red dots) for prolonged stimulations (top). Trial-by-trial identification in derivative space of the deflection points giving origin to the stepwise kinematics (bottom), aligned by the onset of the first movement. High values (yellow colors) indicate sudden acceleration marking the onset of a head motion bout (scale bar: 500 ms, 10 degrees; time bin: 20 ms).

(B) Fraction of trials presenting a given number of steps for progressively longer stimulus duration (data shown in B are examples from one representative animal).

(C–F) Average number of steps (C), step amplitude (D), velocity (E), and duration (F) for increasing stimulus duration across all animals relative to their characteristic vector generated with a 250 ms light stimulus ($n_{TRIALS/MOUSE}$ = 60; n_{MICE} = 12; $p_{AMPLITUDE}$ = 0.60; $p_{VELOCITY}$ = 0.58; $p_{DURATION}$ = 0.06). All results are presented as mean ± SEM. See also Figure S6.

Figure 2C), whose amplitude was consistent from trial to trial. We observed a high degree of correlation between initial and final head-over-body positions following optogenetic stimulation (yaw, R^2 = 0.77; pitch, R^2 = 0.64; roll, R^2 = 0.64), suggesting that activation of Pitx2^{ON} neurons produces a fixed motor program rather than displacements toward a fixed position in space (Figures 2D and 2E). Glutamatergic neurons in the deep and intermediate SC have been also reported to trigger flight responses via a monosynaptic connection to the dorsal periaqueductal gray (dPAG) [19]. However, optogenetic activation of vGlut2^{ON}Pitx2^{ON} neurons at any of the tested stimulation sites, intensities, frequencies, or duration failed to trigger such responses. This is consistent with the absence of projections from Pitx2^{ON} neurons to the dPAG (Figures S3B–S3D) and points, perhaps, to a distinct subclass of glutamatergic collicular neurons as responsible for the described SC-PAG-mediated flight responses.

Having established a causal role of Pitx2^{ON} neurons activity in directing the onset of head movements, we next asked what dictates the exact vectorial nature of the produced displacements. To this aim, we assessed the effect of the modulation of either rate or duration of the optogenetic stimulation of Pitx2^{ON} neurons on the amplitude of the elicited head displacements. Systematic variation of the stimulation frequency between 10 and 50 Hz had no significant impact on the output vector produced (Figures 2F and 2G) [12]. We then analyzed the impact of the duration of stimulation (250 ms to 2 s) on movement amplitude, and we observed that the amplitude of the displacements produced increased with stimulus duration (Figures 2H and 2I; Figures S6F and S6G; Videos S1 and S2). For progressively longer stimulations, however, deflection points could be detected along the trajectory (Figures 3A, 3B, and S6H). Such deflection points represented brief head movements opposite to the ongoing motion, i.e., contraversive, indicating an interrupted attempt to realign head and body position (reset) [37] during the course of prolonged stimulation. These deflection points were nearly equally spaced and appeared at intervals comparable to the displacement vectors produced by the shortest stimulation (referred to as "characteristic vector," Figures 3C–3F; Figures S6I and S6J). The observed stepwise kinematics closely resemble the "staircase" of identical saccades elicited by the electrical stimulation of the SGI in primates [12, 38–40]. Hence, we assessed whether stimulation of Pitx2^{ON} neurons could also elicit eye movements (Figure S7A), as observed in primates following electrical stimulation of the SGI. Indeed, stimulating Pitx2^{ON} neurons with a 250 ms light pulse led to the execution of rapid but very low-amplitude eye movements (<5 degrees) when applied to either the contralateral (Figures S7C, S7F, and S7G) or the ipsilateral hemisphere (Figures S7C, S7F, and S7H), in line with the previously observed bilaterally coordinated eye movements in freely moving rodents [41, 42]. However, unlike what observed for eye movements in primates [12], prolonged stimulations of Pitx2^{ON} neurons (500 ms) failed to elicit a staircase of saccadic eye movement, driving instead monotonic eye movements whose small amplitude remained constant with increased stimulation time (Figures S7B and S7E). Stimulations of Pitx2^{ON} neurons also consistently induced pupil dilation (Figures S7D, S7G, and S7H), suggesting a concomitant increase in arousal. Taken together, these results support the view of a stepwise output of Pitx2^{ON} modules, with respect to head movements, in which progressively longer stimulations produce a concatenated series of fixed characteristic movement vectors.

Pitx2^{ON} Neurons Are Active Prior to Head Displacements of Fixed Metric

While these findings indicate that on exogenous stimulation Pitx2^{ON} neurons are capable of driving head motion, they do

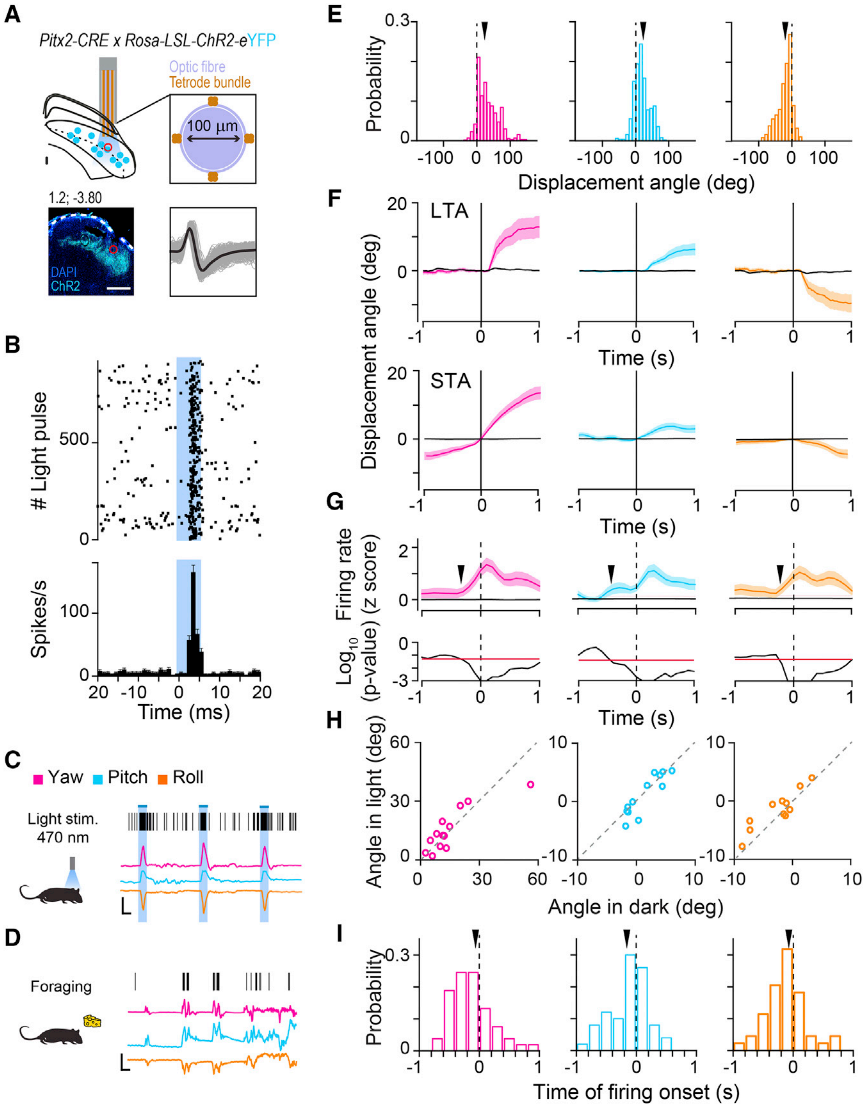

Figure 4. Pitx2^{ON} Neurons Are Active Prior to Head Displacements of Fixed Metric

(A) Optetrode targeted to the SGI of a *Pitx2-CRE::Rosa-LSL-ChR2-eYFP* mouse (top left). Optetrode design (top right): tetrode bundles (gold) around the fiber optic core (blue). Immunohistochemistry showing successful targeting of the optetrode to the SGI (bottom left, scale bar: 500 μm). Spike-sorted waveforms of a responding unit expressing ChR2 (bottom right).

(B) Raster plot (top) and PSTH (bottom) for the single unit in (A) during multiple trials of blue-light stimulation (5 ms, shaded area).

(C) Example of spiking activity from an optotagged unit during blue-light stimulation during open-field explorative behavior, aligned to the simultaneously recorded head displacements in yaw (magenta), pitch (blue), and roll (orange) components (scale bars: 250 ms, 20 degrees).

(D) Same as in (C), for the same unit recorded during foraging behavior in the absence of blue-light stimulation.

(E) Distribution of yaw, pitch, and roll head movements following the spikes of an example optotagged unit. The characteristic angle for each unit was measured as the mean of these distributions (arrowhead).

(F) Light-triggered (LTA, top) and spike-triggered average (STA, bottom) of head movements in the yaw, pitch, and roll components calculated for the optotagged neuron in (E). LTA and STA were tested for significance against shuffled data (black curves).

(G) Average changes in firing rate for the optotagged neuron in (F) around the onset (dashed line) of head movements, calculated separately for yaw, pitch, and roll displacements. The time of firing onset was identified by comparison against null distributions obtained from shuffled data (black arrowhead; red line indicates the significance) (bottom).

(H) For each optotagged unit recorded, the characteristic yaw, pitch, and roll movement angles measured during foraging behavior in the presence of light are plotted against the angles measured during foraging in the dark ($p_{YAW} = 0.89$; $p_{PITCH} = 0.30$; $p_{ROLL} = 0.10$).

(I) Distribution of firing onset across optotagged units with respect to the onset of yaw, pitch, and roll movements. The mean is marked by the arrowhead ($n_{UNITS} = 13$, $n_{MICE} = 2$). All results are presented as mean ± SEM.

See also Figure S4.

not clarify whether Pitx2^{ON} neurons are recruited during spontaneous head movements. Hence, we asked whether spontaneous activity of Pitx2^{ON} neurons was predictive of the onset and the vectorial nature of subsequent head displacements. To do so, we recorded Pitx2^{ON} neurons activity using optetrodes implanted in *Pitx2-Cre::Rosa-LSL-ChR2-eYFP* mice (Figure 4A) during a naturalistic foraging behavior while simultaneously monitoring their head kinematics (Figures 4C and 4D; Figures S4L and S4M). We then isolated Pitx2^{ON} units from our recordings on the basis of the latencies of their responses to light stimulations (<5 ms, Figure 4B). Across multiple recording sessions, Pitx2^{ON} neurons activity was highly correlated to, and predictive of, the produced head displacement vector (Figures 4E, 4F, and 4H), with their firing activity preceding the onset of motion (*Z* score of neural activity significantly increasing prior to motion onset) (Figures 4G and 4I). Moreover, the optetrode approach also allowed us to compare directly, within the same animal and recording site, the amplitude of motion following neural activity (spike-triggered average [STA]), with the amplitude of head movements produced by the optogenetic stimulation (light-triggered average [LTA]). STA and LTA were in the same direction, defining closely matching movement vectors (Figure 4F, $p_{YAW} = 0.55$, $p_{PITCH} = 0.08$, $p_{ROLL} = 0.19$). Overall, these findings indicate a direct and physiological role for Pitx2^{ON} neurons in dictating the vectorial nature of head movements.

Pitx2^{ON} Neurons Define Discrete Modules Tiling the SGI

Having established the role of Pitx2^{ON} neurons in the production of head movements of distinct amplitude and direction, we then analyzed their anatomical distribution and morphological features

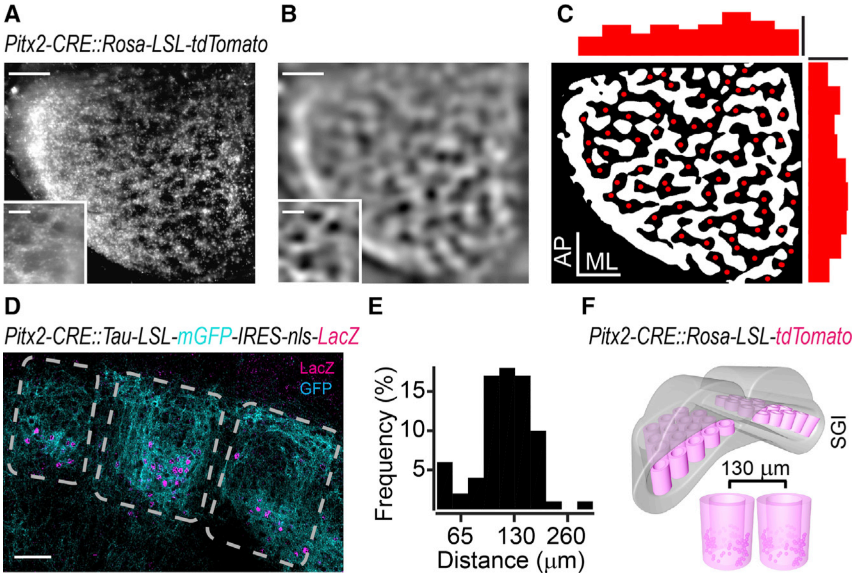

Figure 5. Pitx2^{ON} Neurons Are Organized in Discrete Anatomical Modules in the SGI

(A–C) Projection of top SC view in clarified brain of *Pitx2-CRE::Rosa-LSL-tdTomato* (A), Gaussian filtered image (B), image segmentation and centroid detection of Pitx2^{OFF} zones using the local minima criterion (red dots) and distribution density of Pitx2^{OFF} zone centroids in the medial-lateral (ML) and anterior-posterior (AP) SGI co-ordinates (scale bars = 300 μm; density = 30 centroids/mm^2; (C). Insets represent a magnified top view portion of SC highlighting the Pitx2^{ON} and Pitx2^{OFF} zones with a grid-like organization (scale bar = 130 μm).

(D) Modular organization of Pitx2^{ON} neurons dendrites (cyan, mGFP) and cell bodies (magenta, LacZ) from a coronal section of *Pitx2-CRE::Tau-LSL-mGFP-IRES-nls-LacZ* mice (scale bar: 100 μm).

(E) Distribution of distances of each of the detected Pitx2^{OFF}zone centroids in (C) from the nearest centroid.

(F) Three-dimensional representation of the mouse SC based on clarified brain of *Pitx2-CRE::Rosa-LSL-tdTomato*. The SGI is populated by Pitx2^{ON} neurons (magenta dots represent the cell body) organized in discrete anatomical modules (magenta barrels) the center of which is categorized as a Pitx2^{OFF}space. Pitx2^{OFF}spaces appear as non-fluorescent zones whose centroids distance is 130 ± 7 μm on average (n_{MICE} = 3).

within the SC. To do so, we crossed *Pitx2-Cre* with *Rosa-(LSL)-tdTomato* mice and reconstructed the three-dimensional density of red fluorescent Pitx2^{ON} neurons using light-sheet microscopy in cleared brains. We observed that Pitx2^{ON} neurons were organized in modules, delineating a honeycomb structure characterized by Pitx2^{ON} and Pitx2^{OFF} zones spanning the entire SGI. Transversal maximal projections of Pitx2^{ON} neurons density revealed that the honeycomb spanned the vertical dimension of the SGI (Figures 5A and 5B), with vertical hollow barrels repeating periodically with uniform density along anterior-posterior (AP) and medial-lateral (ML) axis of the SGI (Figure 5C). The spatial constant separating adjacent barrels of the honeycomb was 130 ± 7 μm (Figures 5E and 5F).

In separate experiments, to differentially label Pitx2^{ON} dendrites and somas, we crossed *Pitx2-Cre* with *Tau-LSL-mGFP-nls-LacZ* and processed coronal SC sections for immunohistochemistry against membrane-bound GFP and nuclear LacZ. Both the somas and the dendrites of Pitx2^{ON} neurons respected the same modular organization (Figure 5D); the dendrites of Pitx2^{ON} neurons arborized largely within the SGI within the same parent module of their soma, suggesting that such modularity could be functionally exploited by incoming inputs.

Pitx2^{ON} Modules Define a Motor Map for Head Movements

Given that the output of Pitx2^{ON} neurons in a given SC location appears to be a fixed head movement vector, we tested whether the positional identity of the activated Pitx2^{ON} module was the key determinant of the movement amplitude and direction. Additionally, we assessed the relationship between the produced head displacement vectors and the topography of Pitx2^{ON} modules. To this end, we implanted a custom-made 3×3 multi fiber array in the SGI (Figure 6A; Figure S4K) in *Pitx2-CRE*::*Rosa-LSL-ChR2-eYFP* mice in order to individually stimulate nine collicular sites along the medial-lateral and anterior-posterior axis of the SC of an individual mouse. We then applied a light stimulation pattern consisting of repeating 250 ms pulses through one of the fibers in the array at a time. The estimated illumination area obtained with the optic fibers used was compatible with our measurements of the periodicity defined by *Pitx2* expression in the SGI (Figure 5). This suggests that the results of our optogenetic stimulation could be approximated to the activation of one Pitx2^{ON} module (Figures S4H–S4J), hence revealing whether the positional identity of the stimulated module is a key determinant of the movement vector produced.

Stimulation of distinct collicular sites elicited distinct characteristic movement vectors (Figures 6B–6F), all strictly contralateral to the site of stimulation. Yaw and roll amplitudes increased from anterior to posterior sites, while remaining relatively constant along the medial-lateral axis. Both upward and downward pitch were mapped on a hemi-lateral SC, with upward pitch being represented in the anterior-medial portions of the SC and downward pitch in the posterior-lateral region (Figures 6B–6F).

We further analyzed the head motion data obtained with optogenetic stimulation through the array to estimate the magnification factor [43] for head movement representation in the SC. We observed a uniform magnification factor across the SC, which increased in the anterior-medial part of the SC (Figures 6F and 6G).

These findings were consistent with the retrospective assessment of the distribution of head movement vectors in the SC based on the single optic fiber experiments (Figures 2 and 3), for which all implantation sites were histologically confirmed post hoc for all animals analyzed (Figures S6A–S6E). Overall, these experiments reveal the topographic nature of the Pitx2^{ON} neurons-dependent motor-vector map in the SGI.

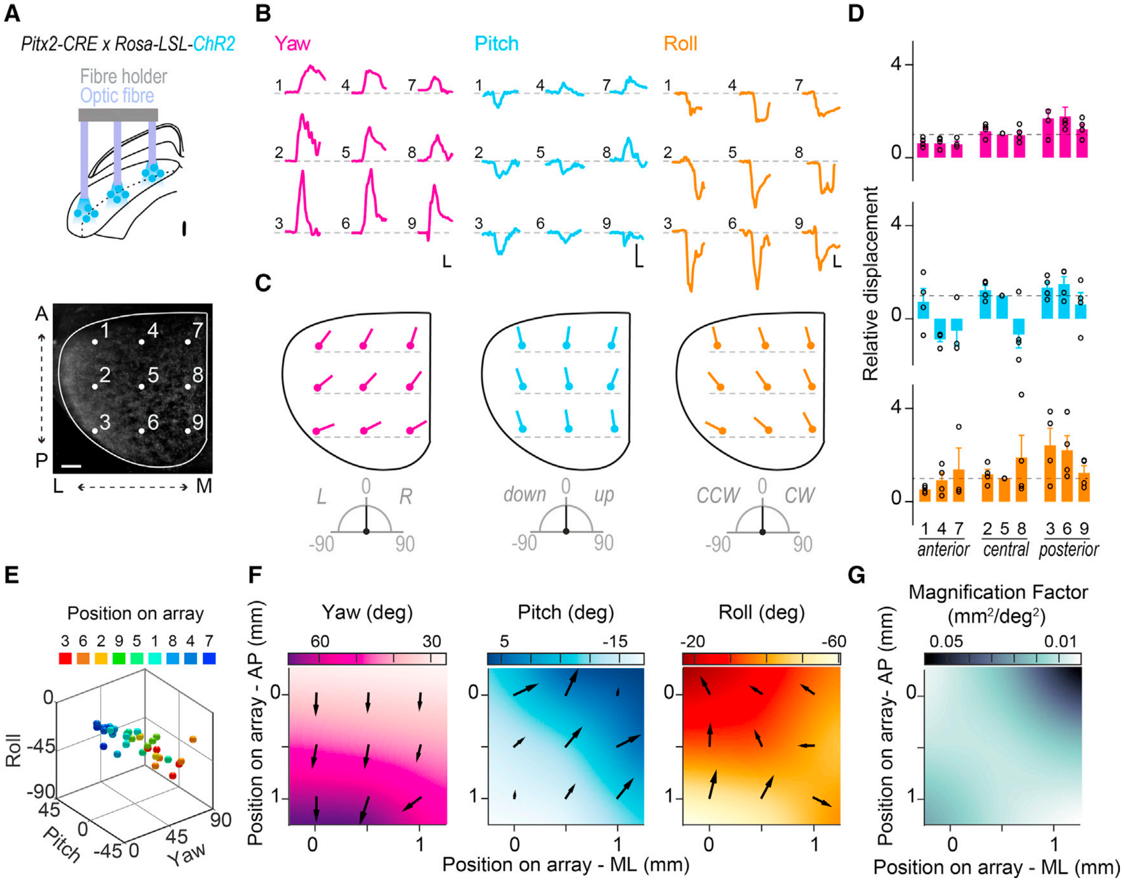

Figure 6. Pitx2^{ON} Modules Define a Motor Map for Head Movements

(A) Schematic of the optic fiber array (top) with fiber reference numbers (center) and schematic representation of array placement in the SC (top view) in a *Pitx2-CRE::Rosa-LSL-tdTomato* clarified brain (scale bar: 250 μm) (bottom).

(B) Representative traces of the light-triggered three-dimensional head movement for each site decomposed in yaw (magenta), pitch (blue), and roll (orange) components (scale bars: 200 ms, 20 degrees).

(C) Average movement vector produced in yaw (left; left [L] and right [R] movements), pitch (center; up or down movements), and roll (right; counter-clockwise [CCW] or clockwise [CW] movements) by light stimulation of nine SC locations from one representative animal shown in (B).

(D) Average amplitude of the light-driven displacement for each site and each Eulerian component, relative to the amplitude of head displacement generated at position 5 within each array ($n_{TRIALS/POSITION} = 40$; $n_{MICE} = 4$). Statistically significant comparisons are, for yaw, Pos1–3 (p = 0.04), Pos1–6 (p = 0.03), Pos3–4 (p = 0.04), Pos4–6 (p = 0.02), Pos6–7 (p = 0.04); for pitch, Pos2–4 (p = 0.03), Pos3–4 (p = 0.02), Pos3–8 (p = 0.04), Pos4–6 (p = 0.01), Pos6–8 (p = 0.03). All results are presented as mean ± SEM.

(E) 3D representation of joint yaw, pitch, and roll movements across the nine SC sites studied. Each sphere represents the average movement vector produced in yaw, pitch, and roll for each animal ($n_{TRIALS/POSITION} = 40$; $n_{MICE} = 4$).

(F) Average movement vectors produced in yaw (magenta), pitch (cyan), and roll (orange) across the nine SC sites represented in color-coded maps. The maps were smoothed and interpolated to emphasize the changes in degrees across the medial-lateral and anterior-posterior SC. Black arrows indicate the direction of local gradient for yaw (left), pitch (center), and roll (right) computed at each location of the array (AP, anterior-posterior; ML, medial-lateral). The length of the arrow indicates the rate of change of the angle over the SC surface.

(G) Magnification factor map computed from the maps of yaw and pitch (in mm^2/deg^2). At each location, the magnification factor indicates how finely angular movements are represented over the SC surface (AP, anterior-posterior; ML, medial-lateral).

See also Figure S4.

Pitx2^{ON} Modules Form a Site of Convergence for Sensory and Motor-Related Information

The execution of spatially tuned actions requires the convergence of multimodal sensory stimuli, as well as of internally generated signals, onto a motor network able to produce appropriate orienting responses. The anatomical modularity of Pitx2^{ON} neuronal cell bodies and dendrites, together with their direct role in dictating the metric of head movements, seems uniquely suited to ensure such a coherent spatial-motor integration of incoming inputs. For this reason, we sought to establish the sources of inputs to Pitx2^{ON} neurons by performing *trans*-synaptic retrograde tracing using monosynaptically restricted Rabies virus [44]. We first injected a FLP-dependent AAV virus expressing Rabies-G protein and TVA receptor (AAV(1)-FRT-RG-2A-TVA-mCherry-2A-Gly) in the SGI of *Pitx2-CRE*::*Tau-LSL-FLPo-INLA* mice followed by the injection of an EnvA-ΔG-Rabies^{GFP} to selectively target TVA-expressing Pitx2^{ON} neurons (Figures 7A and 7B). This approach revealed

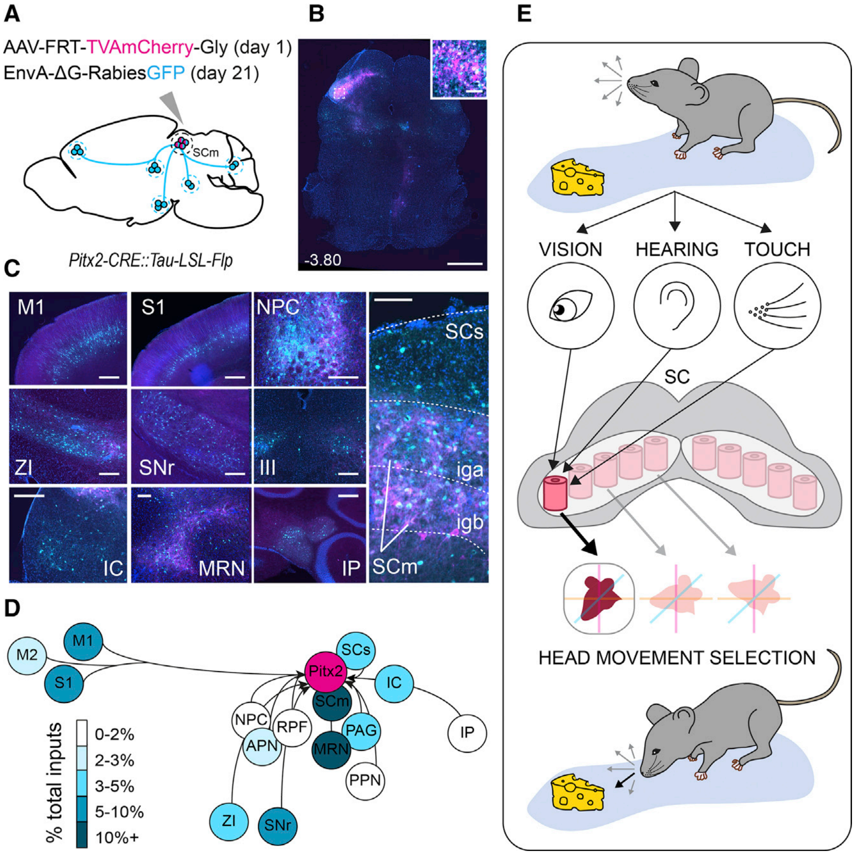

Figure 7. Pitx2^{ON} Modules Form a Site of Convergence for Sensory and Motor-Related Information

(A) Brain-wide pre-synaptic input to Pitx2^{ON} neurons in *Pitx2-CRE::Tau-LSL-FLPo-INLA* mice revealed through the selective targeting of Pitx2^{ON} neurons via a FLP-dependent AAV expressing TVA-Gly (magenta, TVAmCherry) followed by EnvA-ΔG-Rabies^{GFP} (cyan, GFP) injection.

(B and C) Overview of targeted neurons (B) (scale bar: 1 mm, inset: 100 μm) and their presynaptic cortical and subcortical partners (C) (scale bars: 250 μm).

(D) Relative contribution of distinct brain regions to the presynaptic network of Pitx2^{ON} neurons (n_{MICE} = 3). Input areas are M1, primary motor cortex; M2, secondary motor cortex; S1, primary somatosensory cortex; ZI, zona incerta; NPC, nucleus of the posterior commissure; APN, anterior pretectal nucleus; SNr, substantia nigra pars reticulata; III, oculomotor nucleus; SCs, superior colliculus, sensory related; SCm, superior colliculus, motor related; MRN, midbrain reticular nucleus; PAG, periaqueductal gray; PPN, pedunculopontine nucleus; IC, inferior colliculus; IP, interposed nucleus.

(E) Schematic summary of the selection of orienting movements in the mouse SC. Multimodal stimuli (vision, hearing, touch), coming from the environment, converge to recruit specific Pitx2^{ON} neuronal modules in the SGI (pink barrel). The SGI integrates the incoming signals and orchestrates the selection of appropriate Pitx2^{ON} modules according with a motor map (black arrow). Activation of Pitx2^{ON} neurons generates spatially targeted head movements.

See also Figure S3.

multiple sources of input to Pitx2^{ON} neurons, with presynaptic partners located in both cortical and subcortical structures (Figures 7C and 7D; Figures S3E–S3J) in both ipsi- and contralateral hemispheres to the injection site. The vast majority of inputs generated in the ipsilateral hemisphere (84% ± 3%), and midbrain nuclei provided 75% ± 1% of the total input to these cells (Figure S3G).

Our findings suggest a high level of convergence of inputs to Pitx2^{ON} neurons from within the SC (superior colliculus, motor related [SCm], 33% ± 2%, and sensory related [SCs], 6% ± 1%, Figures 7C and 7D; Figure S3F). The presence of inputs from the visual layers of the SC, together with the afferent connections from the somatosensory cortex (S1, 6% ± 1%; Figures 7C and 7D; Figure S3F) and the inferior colliculus (IC, 6% ± 3%; Figures 7C and 7D; Figure S3F), suggests that a degree of multisensory convergence exists on Pitx2^{ON} neurons directly, which might be instrumental to the generation of spatially appropriate motor commands.

A number of motor-related regions were also identified in our retrograde tracing study, among which are midbrain reticular nucleus (MRN, 12% ± 1%; Figures 7C and 7D; Figure S3F), primary motor cortex (M1, 6% ± 3%; Figures 7C and 7D; Figure S3F), and deep cerebellar nuclei, such as the interposed nucleus (IP, 2% ± 1%; Figures 7C and 7D; Figure S3F). Notably, structures previously known to provide a patchwork innervation to the SGI, such as the substantia nigra pars reticulata (SNr, 9% ± 1%; Figures 7C and 7D; Figure S3F) [45] and the pedunculopontine tegmental nucleus (PPN, 2% ± 0.2%; Figures 7C and 7D; Figure S3F) [46, 47], directly synapse onto Pitx2^{ON} neurons, revealing a matching modular design at both the pre- and the post-synaptic levels. These results suggest that the patchwork nature of subcortical projections to the SC obey to a spatial logic, with anatomically segregated afferents selectively recruiting collicular neurons directing movements toward specific spatial coordinates. In particular, the multimodal nature of the monosynaptic inputs to Pitx2^{ON} neurons suggests that they might integrate spatially coherent multisensory, motor, and attentional signals in order to coordinate the production of spatially targeted actions (Figure 7E).

DISCUSSION

One of the most vital tasks that any animal has to face in order to survive is transforming spatial information, as sampled through the senses, into motor plans. Orienting actions rely on the ability to use sensory information available in the surrounding environment and captured by whiskers, eyes, and pinnae, to trigger the appropriate motor output. In rodents, these are primarily represented by head movements, their main ethological orienting behavior. Although the SC has been implicated in the control of such orienting actions, the precise network organization and

associated computations that govern its sensorimotor integration function are yet to be elucidated.

Adopting a genetic approach, we isolated a key neuronal component of this SGI collicular network, we identified its associated pre- and post-synaptic partners, and we investigated its relevance to motor output with particular attention on head movements. Our findings place Pitx2^{ON} neurons at the center of such a spatial motor transformation network. First, we show that Pitx2^{ON} neurons are a homogeneous class of glutamatergic neurons organized in modules tiling the entire SGI. Second, we demonstrate that their activation produces head movements characterized by stereotyped kinematics, whose direction and amplitude varied topographically with the activated module according with a motor map.

The most striking feature of this network is its granularity. Such a modular organization resembles the previously described patchy distribution of the terminal arborizations of subcortical neurons projecting to the SGI, such as of those originating from the SNr and the PPN [46–48]. The geometrical organization of these afferent systems has been proposed to play a role in the selection of movement vectors [26–28]. However, to date, there is no evidence regarding the functional relevance of this anatomical modularity, nor any evidence of the anatomical clustering of SGI neurons according to spatial or motor features. Here, we show that Pitx2^{ON} premotor units in the SGI are also clustered into discrete modules, which are the direct post-synaptic target of these known patchy subcortical afferents. Hence, both input and output systems to the motor SC are arranged in discrete and spatially coherent modules. Such organization provides a site of convergence for multiple cortical and subcortical inputs, supporting the spatially coherent integration of sensory and motor-related information in order to drive orienting responses toward defined portions of space. In essence, Pitx2^{ON} modules act as discrete functional orienting units.

This modularity mirrors the clustered organization of orientation- and direction-selective neurons observed in the visual layers of the murine SC [49, 50], suggesting the existence of a functional modularity for the representation of both sensory and motor information in the SC. There is no *a priori* reason for which motor space should be mapped in functional clusters in the CNS. Yet, modularity can be observed in cortical and subcortical areas subtending motor [51–53], sensory [54–56], and cognitive [57, 58] functions. Such a widespread use of modular designs has been related to principles of wiring economy [59] as well as to the need of constraining synaptic connectivity in an anatomically selective manner [56, 58, 60]. Here, we favor the latter and propose that modularity of Pitx2^{ON} modules gives origin to a discrete spatial-motor register addressable by cortical and subcortical inputs for the execution of accurate and precise voluntary actions toward desired positions in space.

We also uncovered multiple similarities between the principles governing the control of head displacement vectors in mice and those controlling saccadic eye movements in primates. These cross-species similarities suggest that the computational logic governing gaze shifts in primates and cats [11, 61–63] is primarily implemented, in afoveated species such as mice, for the control of the head movement component. Indeed, the extremely low amplitude of the eye movements elicited by Pitx2^{ON} neurons stimulation and the lack of any observable stepwise kinematics upon prolonged stimulation regimes suggest that Pitx2^{ON} neurons-mediated eye movements are qualitatively different from canonical saccades observed in primates. They might represent instead vestibular-independent anticipatory compensatory responses to the forthcoming head motion. Efferent copy signals related to voluntary head movements have been previously observed both in rodents and monkeys [64–69], and they have been shown to drive the execution of anticipatory eye movements with a gaze-stabilizing function [64, 66, 70].

Overall, we interpret these results as evidence that collicular circuit function has evolved to solve the general task of transforming spatial information into appropriate movement vectors and to do so in a way that is most ethologically relevant for the species. In the case of rodents, which possess afoveated eyes, gaze is nearly entirely the product of head rotations and hence collicular circuits prioritize the representation of these motor plans for orienting. These similarities suggest the existence of an evolutionarily conserved genetic program shaping primates' and rodents' gaze-control circuits, albeit with species-specific weights of the relative head and eye components on the final gaze output, raising the possibility that Pitx2^{ON} neurons are involved in the control of the full metric of gaze in humans and non-human primates. This speculation receives at least some support from gene expression data revealing the conserved collicular expression of *Pitx2* in humans [71, 72].

In line with this idea of the universal role of the SC in spatial reorienting, it has been suggested that the primary overarching function of the SC is to act as a global spatial indexing system [73], whose function would be to pull together spatially coherent signals from different brain regions in order to direct overt and covert spatially tuned responses. Our results support this model and, while in this report we only characterized the role of Pitx2^{ON} modules in the execution of overt head reorienting movements, the concomitant pupil dilation observed upon optogenetic activation of Pitx2^{ON} neurons suggests their possible involvement also in spatially tuned covert attentional responses.

Overall, our results suggest the presence of a functional map of head movements that resembles the motor map of saccades measured in cats and primates [14], suggesting an evolutionary conserved neuronal basis of spatially tuned orienting behaviors. Even if we are still far from fully appreciating how different neuronal types operate together to interpret the incoming sensory flow and convey this information in a meaningful manner to the motor domain, with the adopted genetic-based reconstruction of collicular circuits, we provide an entry point for future investigation on the network principle of spatial-motor transformations in the mouse SC.

STAR★METHODS

Detailed methods are provided in the online version of this paper and include the following:

- KEY RESOURCES TABLE
- LEAD CONTACT AND MATERIALS AVAILABILITY
- EXPERIMENTAL MODEL AND SUBJECT DETAILS

- METHOD DETAILS
  - *In vitro* electrophysiology
  - RNA extraction and cDNA library preparation
  - Immunohistochemistry
  - *In situ* hybridization
  - Whole-brain clarification and imaging
  - Surgical procedures
  - Viral injections
  - 3D tracking of head-over-body position
  - Optogenetic stimulation in freely moving animals
  - Optogenetic stimulation in head-restrained animals
  - *In vivo* electrophysiology
- QUANTIFICATION AND STATISTICAL ANALYSIS
  - *In vitro* electrophysiology
  - RNA-seq data processing
  - Histological analysis
  - Light sheet microscopy imaging analysis
  - Characterization of 3D head movements
  - *In vivo* optogenetics
  - Eye tracking
  - Spike sorting
  - Determination of motion tuning
  - Spike and light triggered average
  - Generation of shuffled datasets
  - Statistical methods
- DATA AND CODE AVAILABILITY

SUPPLEMENTAL INFORMATION

Supplemental Information can be found online at https://doi.org/10.1016/j.cub.2019.07.083.

ACKNOWLEDGMENTS

We would like to thank Michael Hastings, Gregory Jefferis, Silvia Arber, Andreas Kardamakis, and Kathleen Cullen for comments on the manuscript; Michael Krumin and Matteo Carandini for providing the code for the eye movements' analysis; Ana Gonzalez Rueda and Jonathan Wilson for helping with setting up the eye tracking system and the analysis routines; and Ernesto Ciabatti for the design of the viral vectors. We would like to thank Silvia Arber for sharing with us the *Tau-LSL-FLPo-INLA* and *Tau-LSL-mGFP-INLA* mice. We thank the Laboratory of Molecular Biology (LMB) electronics and mechanical workshops for the help with hardware development, members of the Biological Service Group for their support with the *in vivo* animal work, and the LMB Light Microscopy group for helping with the acquisition and the analysis of imaging data. This study was supported by the Medical Research Council (MC_UP_1201/2), the European Research Council with an ERC Starting Grant to M.T. (STG 677029), and the ERANET-NEURON (Micronet) grant to M.T.

AUTHOR CONTRIBUTIONS

M.T. initiated the project and performed the preliminary experiments. M.T., L. Masullo, and L. Mariotti designed the experiments. L. Masullo and L. Mariotti performed the *in vitro* recordings. L. Masullo performed the optical stimulation experiments with the help of L. Mariotti. L. Mariotti performed the *in vivo* optetrodes recordings with the help of L. Masullo. L. Masullo performed the viral circuit mapping experiments, the genetic screening, and the molecular phenotyping. P.F.-P. and L. Masullo analyzed the deep sequencing data. L. Masullo, L. Mariotti, and J.B. analyzed the imaging data. M.T. and N.A. designed hardware and software with L. Mariotti and L. Masullo. L. Masullo, L. Mariotti, M.T., and N.A. analyzed the data. L. Masullo, L. Mariotti, and M.T. prepared the figures. M.T. wrote the manuscript with L. Masullo and L. Mariotti incorporating feedback from N.A.

DECLARATION OF INTERESTS

The authors declare no competing interests.

Received: June 3, 2019
Revised: July 26, 2019
Accepted: July 30, 2019
Published: August 29, 2019

REFERENCES

1. Graziano, M.S., Hu, X.T., and Gross, C.G. (1997). Coding the locations of objects in the dark. Science *277*, 239–241.
2. Fogassi, L., Gallese, V., di Pellegrino, G., Fadiga, L., Gentilucci, M., Luppino, G., Matelli, M., Pedotti, A., and Rizzolatti, G. (1992). Space coding by premotor cortex. Exp. Brain Res. *89*, 686–690.
3. Stanford, T.R., Quessy, S., and Stein, B.E. (2005). Evaluating the operations underlying multisensory integration in the cat superior colliculus. J. Neurosci. *25*, 6499–6508.
4. Calvert, G., Spence, C., and Stein, B.E. (2004). The Handbook of Multisensory Processes (MIT Press).
5. Meredith, M.A. (2002). On the neuronal basis for multisensory convergence: a brief overview. Brain Res. Cogn. Brain Res. *14*, 31–40.
6. May, P.J. (2006). The mammalian superior colliculus: laminar structure and connections. Prog. Brain Res. *151*, 321–378.
7. Wallace, M.T., Meredith, M.A., and Stein, B.E. (1998). Multisensory integration in the superior colliculus of the alert cat. J. Neurophysiol. *80*, 1006–1010.
8. Wallace, M.T., Meredith, M.A., and Stein, B.E. (1993). Converging influences from visual, auditory, and somatosensory cortices onto output neurons of the superior colliculus. J. Neurophysiol. *69*, 1797–1809.
9. Gharaei, S., Arabzadeh, E., and Solomon, S.G. (2018). Integration of visual and whisker signals in rat superior colliculus. Sci. Rep. *8*, 16445.
10. Sparks, D.L. (1999). Conceptual issues related to the role of the superior colliculus in the control of gaze. Curr. Opin. Neurobiol. *9*, 698–707.
11. Munoz, D.P., Guitton, D., and Pélisson, D. (1991). Control of orienting gaze shifts by the tectoreticulospinal system in the head-free cat. III. Spatiotemporal characteristics of phasic motor discharges. J. Neurophysiol. *66*, 1642–1666.
12. Schiller, P.H., and Stryker, M. (1972). Single-unit recording and stimulation in superior colliculus of the alert rhesus monkey. J. Neurophysiol. *35*, 915–924.
13. Munoz, D.P., Pélisson, D., and Guitton, D. (1991). Movement of neural activity on the superior colliculus motor map during gaze shifts. Science *251*, 1358–1360.
14. Gandhi, N.J., and Katnani, H.A. (2011). Motor functions of the superior colliculus. Annu. Rev. Neurosci. *34*, 205–231.
15. Wurtz, R.H., and Albano, J.E. (1980). Visual-motor function of the primate superior colliculus. Annu. Rev. Neurosci. *3*, 189–226.
16. Stein, B.E., and Clamann, H.P. (1981). Control of pinna movements and sensorimotor register in cat superior colliculus. Brain Behav. Evol. *19*, 180–192.
17. Dean, P., Redgrave, P., Sahibzada, N., and Tsuji, K. (1986). Head and body movements produced by electrical stimulation of superior colliculus in rats: effects of interruption of crossed tectoreticulospinal pathway. Neuroscience *19*, 367–380.
18. Dean, P., Redgrave, P., and Mitchell, I.J. (1988). Organisation of efferent projections from superior colliculus to brainstem in rat: evidence for functional output channels. Prog. Brain Res. *75*, 27–36.
19. Evans, D.A., Stempel, A.V., Vale, R., Ruehle, S., Lefler, Y., and Branco, T. (2018). A synaptic threshold mechanism for computing escape decisions. Nature *558*, 590–594.

20. Moschovakis, A.K., Scudder, C.A., and Highstein, S.M. (1996). The microscopic anatomy and physiology of the mammalian saccadic system. Prog. Neurobiol. *50*, 133–254.

21. Saito, Y., and Isa, T. (1999). Electrophysiological and morphological properties of neurons in the rat superior colliculus. I. Neurons in the intermediate layer. J. Neurophysiol. *82*, 754–767.

22. Sooksawate, T., Isa, K., Matsui, R., Kato, S., Kinoshita, M., Kobayashi, K., Watanabe, D., Kobayashi, K., and Isa, T. (2013). Viral vector-mediated selective and reversible blockade of the pathway for visual orienting in mice. Front. Neural Circuits *7*, 162.

23. Wilson, J.J., Alexandre, N., Trentin, C., and Tripodi, M. (2018). Three-Dimensional Representation of Motor Space in the Mouse Superior Colliculus. Curr. Biol. *28*, 1744–1755.e12.

24. Felsen, G., and Mainen, Z.F. (2008). Neural substrates of sensory-guided locomotor decisions in the rat superior colliculus. Neuron *60*, 137–148.

25. Semina, E.V., Reiter, R., Leysens, N.J., Alward, W.L., Small, K.W., Datson, N.A., Siegel-Bartelt, J., Bierke-Nelson, D., Bitoun, P., Zabel, B.U., et al. (1996). Cloning and characterization of a novel bicoid-related homeobox transcription factor gene, RIEG, involved in Rieger syndrome. Nat. Genet *14*, 392–399.

26. Illing, R.B., and Graybiel, A.M. (1985). Convergence of afferents from frontal cortex and substantia nigra onto acetylcholinesterase-rich patches of the cat's superior colliculus. Neuroscience *14*, 455–482.

27. Mana, S., and Chevalier, G. (2001). Honeycomb-like structure of the intermediate layers of the rat superior colliculus: afferent and efferent connections. Neuroscience *103*, 673–693.

28. Mana, S., and Chevalier, G. (2001). The fine organization of nigro-collicular channels with additional observations of their relationships with acetylcholinesterase in the rat. Neuroscience *106*, 357–374.

29. Oliveira, A.F., and Yonehara, K. (2018). The Mouse Superior Colliculus as a Model System for Investigating Cell Type-Based Mechanisms of Visual Motor Transformation. Front. Neural Circuits *12*, 59.

30. Arthur, D., and Vassilvitskii, S. (2007). k-means plus plus: The Advantages of Careful Seeding. Proceedings of the Eighteenth Annual Acm-Siam Symposium on Discrete Algorithms, 1027–1035.

31. Madisen, L., Zwingman, T.A., Sunkin, S.M., Oh, S.W., Zariwala, H.A., Gu, H., Ng, L.L., Palmiter, R.D., Hawrylycz, M.J., Jones, A.R., et al. (2010). A robust and high-throughput Cre reporting and characterization system for the whole mouse brain. Nat. Neurosci. *13*, 133–140.

32. Vong, L., Ye, C., Yang, Z., Choi, B., Chua, S., Jr., and Lowell, B.B. (2011). Leptin action on GABAergic neurons prevents obesity and reduces inhibitory tone to POMC neurons. Neuron *71*, 142–154.

33. Liu, W., Selever, J., Lu, M.F., and Martin, J.F. (2003). Genetic dissection of Pitx2 in craniofacial development uncovers new functions in branchial arch morphogenesis, late aspects of tooth morphogenesis and cell migration. Development *130*, 6375–6385.

34. Pivetta, C., Esposito, M.S., Sigrist, M., and Arber, S. (2014). Motor-circuit communication matrix from spinal cord to brainstem neurons revealed by developmental origin. Cell *156*, 537–548.

35. Kohl, J., Babayan, B.M., Rubinstein, N.D., Autry, A.E., Marin-Rodriguez, B., Kapoor, V., Miyamishi, K., Zweifel, L.S., Luo, L., Uchida, N., and Dulac, C. (2018). Functional circuit architecture underlying parental behaviour. Nature *556*, 326–331.

36. Madisen, L., Mao, T., Koch, H., Zhuo, J.M., Berenyi, A., Fujisawa, S., Hsu, Y.W., Garcia, A.J., 3rd, Gu, X., Zanella, S., et al. (2012). A toolbox of Cre-dependent optogenetic transgenic mice for light-induced activation and silencing. Nat. Neurosci. *15*, 793–802.

37. Kardamakis, A.A., and Moschovakis, A.K. (2006). Implications of interrupted eye-head gaze shifts for resettable integrator reset. Brain Res. Bull. *70*, 171–178.

38. Robinson, D.A. (1972). Eye movements evoked by collicular stimulation in the alert monkey. Vision Res. *12*, 1795–1808.

39. Breznen, B., Lu, S.M., and Gnadt, J.W. (1996). Analysis of the step response of the saccadic feedback: system behavior. Exp. Brain Res. *111*, 337–344.

40. Missal, M., Lefèvre, P., Delinte, A., Crommelinck, M., and Roucoux, A. (1996). Smooth eye movements evoked by electrical stimulation of the cat's superior colliculus. Exp. Brain Res. *107*, 382–390.

41. Meyer, A.F., Poort, J., O'Keefe, J., Sahani, M., and Linden, J.F. (2018). A Head-Mounted Camera System Integrates Detailed Behavioral Monitoring with Multichannel Electrophysiology in Freely Moving Mice. Neuron *100*, 46–60.e7.

42. Wallace, D.J., Greenberg, D.S., Sawinski, J., Rulla, S., Notaro, G., and Kerr, J.N. (2013). Rats maintain an overhead binocular field at the expense of constant fusion. Nature *498*, 65–69.

43. Garrett, M.E., Nauhaus, I., Marshel, J.H., and Callaway, E.M. (2014). Topography and areal organization of mouse visual cortex. J. Neurosci. *34*, 12587–12600.

44. Stepien, A.E., Tripodi, M., and Arber, S. (2010). Monosynaptic rabies virus reveals premotor network organization and synaptic specificity of cholinergic partition cells. Neuron *68*, 456–472.

45. Graybiel, A.M. (1978). Organization of the nigrotectal connection: an experimental tracer study in the cat. Brain Res. *143*, 339–348.

46. Huerta, M.F., and Harting, J.K. (1982). The projection from the nucleus of the posterior commissure to the superior colliculus of the cat: patch-like endings within the intermediate and deep grey layers. Brain Res. *238*, 426–432.

47. Graybiel, A.M. (1978). A stereometric pattern of distribution of acetylthiocholinesterase in the deep layers of the superior colliculus. Nature *272*, 539–541.

48. Huerta, M.F., Frankfurter, A.J., and Harting, J.K. (1981). The trigeminocollicular projection in the cat: patch-like endings within the intermediate gray. Brain Res. *211*, 1–13.

49. Feinberg, E.H., and Meister, M. (2015). Orientation columns in the mouse superior colliculus. Nature *519*, 229–232.

50. de Malmazet, D., Kühn, N.K., and Farrow, K. (2018). Retinotopic Separation of Nasal and Temporal Motion Selectivity in the Mouse Superior Colliculus. Curr. Biol. *28*, 2961–2969.e4.

51. Georgopoulos, A.P., Merchant, H., Naselaris, T., and Amirikian, B. (2007). Mapping of the preferred direction in the motor cortex. Proc. Natl. Acad. Sci. USA *104*, 11068–11072.

52. Tripodi, M., Stepien, A.E., and Arber, S. (2011). Motor antagonism exposed by spatial segregation and timing of neurogenesis. Nature *479*, 61–66.

53. Aflalo, T.N., and Graziano, M.S. (2006). Possible origins of the complex topographic organization of motor cortex: reduction of a multidimensional space onto a two-dimensional array. J. Neurosci. *26*, 6288–6297.

54. Cohen, Y.E., and Knudsen, E.I. (1995). Binaural tuning of auditory units in the forebrain archistriatal gaze fields of the barn owl: local organization but no space map. J. Neurosci. *15*, 5152–5168.

55. Maruoka, H., Nakagawa, N., Tsuruno, S., Sakai, S., Yoneda, T., and Hosoya, T. (2017). Lattice system of functionally distinct cell types in the neocortex. Science *358*, 610–615.

56. Woolsey, T.A., and Van der Loos, H. (1970). The structural organization of layer IV in the somatosensory region (SI) of mouse cerebral cortex. The description of a cortical field composed of discrete cytoarchitectonic units. Brain Res. *17*, 205–242.

57. Stensola, H., Stensola, T., Solstad, T., Frøland, K., Moser, M.B., and Moser, E.I. (2012). The entorhinal grid map is discretized. Nature *492*, 72–78.

58. Burgalossi, A., Herfst, L., von Heimendahl, M., Förste, H., Haskic, K., Schmidt, M., and Brecht, M. (2011). Microcircuits of functionally identified neurons in the rat medial entorhinal cortex. Neuron *70*, 773–786.

59. Chklovskii, D.B., Schikorski, T., and Stevens, C.F. (2002). Wiring optimization in cortical circuits. Neuron *34*, 341–347.

60. Ji, W., Gămănuţ, R., Bista, P., D'Souza, R.D., Wang, Q., and Burkhalter, A. (2015). Modularity in the Organization of Mouse Primary Visual Cortex. Neuron *87*, 632–643.

61. Paré, M., Crommelinck, M., and Guitton, D. (1994). Gaze shifts evoked by stimulation of the superior colliculus in the head-free cat conform to the motor map but also depend on stimulus strength and fixation activity. Exp. Brain Res. *101*, 123–139.

62. Roucoux, A., Guitton, D., and Crommelinck, M. (1980). Stimulation of the superior colliculus in the alert cat. II. Eye and head movements evoked when the head is unrestrained. Exp. Brain Res. *39*, 75–85.

63. Walton, M.M., Bechara, B., and Gandhi, N.J. (2007). Role of the primate superior colliculus in the control of head movements. J. Neurophysiol. *98*, 2022–2037.

64. Shanidze, N., Kim, A.H., Loewenstein, S., Raphael, Y., and King, W.M. (2010). Eye-head coordination in the guinea pig II. Responses to self-generated (voluntary) head movements. Exp. Brain Res. *205*, 445–454.

65. King, W.M., and Shanidze, N. (2011). Anticipatory eye movements stabilize gaze during self-generated head movements. Ann. N Y Acad. Sci. *1233*, 219–225.

66. Dichgans, J., Bizzi, E., Morasso, P., and Tagliasco, V. (1973). Mechanisms underlying recovery of eye-head coordination following bilateral labyrinthectomy in monkeys. Exp. Brain Res. *18*, 548–562.

67. Cullen, K.E., and Roy, J.E. (2004). Signal processing in the vestibular system during active versus passive head movements. J. Neurophysiol. *91*, 1919–1933.

68. Roy, J.E., and Cullen, K.E. (2001). Selective processing of vestibular reafference during self-generated head motion. J. Neurosci. *21*, 2131–2142.

69. Bizzi, E., Kalil, R.E., and Tagliasco, V. (1971). Eye-head coordination in monkeys: evidence for centrally patterned organization. Science *173*, 452–454.

70. Shanidze, N., Kim, A.H., Raphael, Y., and King, W.M. (2010). Eye-head coordination in the guinea pig I. Responses to passive whole-body rotations. Exp. Brain Res. *205*, 395–404.

71. Hawrylycz, M.J., Lein, E.S., Guillozet-Bongaarts, A.L., Shen, E.H., Ng, L., Miller, J.A., van de Lagemaat, L.N., Smith, K.A., Ebbert, A., Riley, Z.L., et al. (2012). An anatomically comprehensive atlas of the adult human brain transcriptome. Nature *489*, 391–399.

72. Miller, J.A., Ding, S.L., Sunkin, S.M., Smith, K.A., Ng, L., Szafer, A., Ebbert, A., Riley, Z.L., Royall, J.J., Aiona, K., et al. (2014). Transcriptional landscape of the prenatal human brain. Nature *508*, 199–206.

73. Krauzlis, R.J., Lovejoy, L.P., and Zénon, A. (2013). Superior colliculus and visual spatial attention. Annu. Rev. Neurosci. *36*, 165–182.

74. Dobin, A., Davis, C.A., Schlesinger, F., Drenkow, J., Zaleski, C., Jha, S., Batut, P., Chaisson, M., and Gingeras, T.R. (2013). STAR: ultrafast universal RNA-seq aligner. Bioinformatics *29*, 15–21.

75. Love, M.I., Huber, W., and Anders, S. (2014). Moderated estimation of fold change and dispersion for RNA-seq data with DESeq2. Genome Biol. *15*, 550.

76. Susaki, E.A., Tainaka, K., Perrin, D., Yukinaga, H., Kuno, A., and Ueda, H.R. (2015). Advanced CUBIC protocols for whole-brain and whole-body clearing and imaging. Nat. Protoc. *10*, 1709–1727.

77. Anikeeva, P., Andalman, A.S., Witten, I., Warden, M., Goshen, I., Grosenick, L., Gunaydin, L.A., Frank, L.M., and Deisseroth, K. (2011). Optetrode: a multichannel readout for optogenetic control in freely moving mice. Nat. Neurosci. *15*, 163–170.

STAR★METHODS

KEY RESOURCES TABLE

REAGENT or RESOURCE	SOURCE	IDENTIFIER
Antibodies		
Chicken polyclonal anti-GFP	Aves Labs	Cat#: GFP-1020; RRID: AB_10000240
Rabbit polyclonal anti-RFP	Rockland	Cat#: 600-401-379; RRID: AB_2209751
Mouse monoclonal anti-NeuN	Millipore	Cat#: MAB377; RRID: AB_2298772
Chicken polyclonal anti-β-Galactosidase	Abcam	Cat#: ab9361; RRID: AB_307210
Alexa Fluor 488 donkey anti-chicken	Jackson ImmunoResearch	Cat#: 703-545-155; RRID: AB_2340375
Cy3 donkey anti-rabbit	Jackson ImmunoResearch	Cat#: 711-165-152; RRID: AB_2307443
Cy5 donkey anti-mouse	Jackson ImmunoResearch	Cat#: 715-175-150; RRID: AB_2340819
Bacterial and Virus Strains		
AAV(1)-CMV-FRT-TVAmCherry-2A-Gly	Tripodi Lab	N/A
AAV(9)-CMV-FRT-SynGFP-WPRE	Tripodi Lab	N/A
EnvA-ΔG-Rabies^{GFP}	Tripodi Lab	N/A
Chemicals, Peptides, and Recombinant Proteins		
Triethanolamine	Sigma-Aldrich	Cat#: 90279
N,*N*,*N′*,*N′*-Tetrakis(2-Hydroxypropyl)ethylenediamine	Sigma-Aldrich	Cat#: 122262
Platinum Chloride	Sigma Aldrich	Cat#: 206091
Silicon oil	Sigma Aldrich	Cat#: 175633
Mineral oil	Sigma Aldrich	Cat#: M8410
Critical Commercial Assays		
TruSeq® Stranded Total RNA Library Prep Kit	Illumina	Cat#: RS-122-2101
Deposited Data		
RNaseq raw data	This paper	GEO: GSE135082
Experimental Models: Cell Lines		
Mouse: Slc32a1tm2(cre)Lowl	The Jackson Laboratory	RRID:IMSR_JAX:016962
Mouse: Slc17a6tm2(cre)Lowl	The Jackson Laboratory	RRID:IMSR_JAX:016963
Mouse: C57BL/6JOlaHsd	Envigo	Order Code: 057
Mouse: Gt(ROSA)26Sortm14(CAG tdTomato)	The Jackson Laboratory	RRID:IMSR_JAX:007914
Mouse: 129OlaE14CB6(TAU-LSL-MGFP-INLA#18)	The Jackson Laboratory	RRID:IMSR_JAX:021162
Mouse: B6.Cg-Gt(ROSA)26Sortm32 (CAG- COP4*H134R/ EYFP)Hze/J	The Jackson Laboratory	RRID:IMSR_JAX:024109
Mouse: *Tau-LoxP-STOP-LoxP-FLPo-IRES-nLacZ*	[34]	Obtained from Silvia Arber Lab
Mouse: *Pitx2-CRE*	[25]	Obtained from James Martin Lab
Recombinant DNA		
pAAV-CMV-FRT-TVA-mCherry-2A-Gly	Tripodi Lab	Addgene ID: 131342
pAAV-CMV-FRT-TVA-synGFP-WPRE	Tripodi Lab	Addgene ID: 131344
Software and Algorithms		
MATLAB (R2014b)	Mathworks	https://uk.mathworks.com/products/matlab.html
Python 2.7	Continuum Analytics	https://www.anaconda.com
GraphPad Prism7	GraphPad	https://www.graphpad.com/scientific-software/prism/
R (version 3.5.0)	The R project	https://www.r-project.org
STAR_2.5.3a_modified	[74]	https://github.com/alexdobin/STAR/
DESeq2	[75]	https://www.bioconductor.org/packages//2.12/bioc/html/DESeq2.html

(*Continued on next page*)

Continued		
REAGENT or RESOURCE	SOURCE	IDENTIFIER
NIS-Elements HCA 4.30	Nikon	https://www.microscope.healthcare.nikon.com/products/software/nis-elements/nis-elements-hc
Custom Python scripts	This paper	N/A
Custom MATLAB scripts	This paper	N/A
TINT	Axona	Cat#: comp/TINT01, https://www.axona.com/products
Arduino	Arduino	https://www.arduino.cc
Processing 3	Processing	https://processing.org
ImageJ (Fiji 1.48)	Fiji	https://fiji.sc
Other		
Microwire (17 μm, platinum iridium)	California Wire Company	Cat#: 100167
NanoZ plating equipment	Multichannel Systems	nanoZ, https://www.multichannelsystems.com/products/nanoz
Recording system (pre-amp and system unit)	Axona	Cat#: DacqUSB/32,
Mono Fiberoptic Cannula	Doric Lenses Inc.	Cat#: MFC_100/125-0.22_30mm_MF1.25_FLT
Fiberoptic array	Doric Lenses Inc.	Custom part

LEAD CONTACT AND MATERIALS AVAILABILITY

Further information and requests for resources and reagents should be directed to and will be fulfilled by the Lead Contact, Marco Tripodi (mtripodi@mrc-lmb.cam.ac.uk). No mouse lines have been generated for this study. Plasmids used in this study have been deposited to Addgene (IDs: 131342 and 131344).

EXPERIMENTAL MODEL AND SUBJECT DETAILS

Male mice aged between 8 and 12 weeks from of the following lines were used: C57BL/6 wild-type (WT), *vGAT-CRE* (Jackson: Slc32a1tm2(cre)Lowl), *vGluT2-CRE* (Jackson: Slc17a6tm2(cre)Lowl), *Pitx2-CRE* (obtained from James Martin Lab), *Rosa-LoxP-STOP-LoxP-tdTomato* (Jackson: Gt(ROSA)26Sortm14(CAG tdTomato)), *Tau-LoxP-STOP-LoxP-mGFP- IRES-nLacZ* (Jackson: 129OlaE14CB6(TAU-LSL-MGFP-INLA#18)), *Rosa-LoxP-STOP-LoxP-ChR2-eYFP* (Jackson: B6.Cg-Gt(ROSA)26Sortm32(CAG- COP4* H134R/ EYFP)Hze/J), *Tau-LoxP-STOP-LoxP-FLPo-IRES-nLacZ* (obtained from Silvia Arber Lab). All transgenic mice were isogenic in a C57BL/6 background, maintained in pathogen and opportunistic agents-free conditions and monitored quarterly. All procedures were conducted in accordance with the UK Animals (Scientific procedures) Act 1986 and European Community Council Directive on Animal Care. Animals were housed in a 12 hours light/dark cycle with food and water *ad libitum*.

METHOD DETAILS

In vitro electrophysiology

For *in vitro* electrophysiological recordings, C57BL/6 wild-type (WT), vGluT2-CRE::Rosa-LoxP-STOP-LoxP-tdTomato, vGAT-CRE::Rosa-LoxP-STOP-LoxP-tdTomato, Pitx2-CRE::Rosa-LoxP-STOP-LoxP-tdTomato and Pitx2-CRE::Rosa-LoxP-STOP-LoxP-tdTomato::Rosa-LoxP-STOP-LoxP-ChR2-eYFP mice were used. Coronal collicular slices (300 μm) from 2 months old mice were prepared using a vibrating microtome (7000smz-2, Campden Instruments LTD, Loughborough, UK). Animals were anesthetized with 2% isoflurane, then the brain was extracted and immediately transferred in ice-cold solution (aCSF, in mM: 125 NaCl, 2.5 KCl, 2 $CaCl_2$, 1 $MgCl_2$, 25 glucose, pH 7.4 with 95% O_2, and 5% CO_2). Slices were cut in a potassium D-gluconate solution (in mM: 130 potassium gluconate, 15 KCl, 0.2 EGTA, 20 HEPES, 25 glucose, 2 kynurenic acid, to pH 7.4 with NaOH and maintained with 95% O_2, and 5% CO_2) and then kept for 1 minute in a D-mannitol based solution (in mM: 225 D-mannitol, 2.5 KCl, 1.25 NaH_2PO_4, 26 $NaHCO_3$, 25 glucose, 0.8 $CaCl_2$, 8 $MgCl_2$, 2 kynurenic acid with 95% O_2, and 5% CO_2). Finally, slices were incubated in aCSF at 30 °C for 20 minutes and then maintained at RT for the entire experiment. Slices were individually transferred to the recording chamber and perfused with recording solution (in mM: 120 NaCl, 2.5 KCl, 1 NaH_2PO_4, 26 $NaHCO_3$, 1 $MgCl_2$, 2 $CaCl_2$, 10 glucose, pH 7.4 with 95% O_2 and 5% CO_2) at a flow rate of approximately 2 mL/min. Whole-cell patch-clamp recordings were obtained from SC neurons using 8-10 MΩ pipettes pulled from borosilicate glass capillaries (1.5 mm OD x 0.86 mm ID; Harvard Apparatus, Holliston, MA). Pipettes were filled with artificial intracellular solution containing (in mM): 145 potassium gluconate, 5 $MgCl_2$, 0.5 EGTA, 2 Na_2ATP, 0.2 Na_2GTP, 10 HEPES, to pH 7.2 with KOH, osmolarity 280 ÷ 290 mOsm. Single-cell recordings were performed in current-clamp configuration using an Axon Multiclamp 700B amplifier (Molecular Devices, Union City, CA). Signals were low-pass filtered at

2 kHz and acquired at 5 kHz using a digitizer (Axon Digidata 1550A, Molecular Devices, Union City, CA, USA) on a PC running pClamp. Full-field photo-stimulation of ChR2-expressing neurons was done as follows: for continuous light stimulations, we used 0.25, 0.5, 1 or 2 s light pulses; for light pulse stimulations, we used 5 ms light pulses at 10, 20, 30, 40 or 50 Hz. Light-evoked responses were elicited using a 450-490 nm LED (pE-300 coolLED system, Scientifica Ltd, Uckfield, UK) through a 40x water immersion objective (LUMPlan FI/IR 40 × , 0.8NA, Olympus, Tokyo, Japan). Access resistance was monitored throughout the recordings and was between 16.66 and 42.22 MΩ. Neurons that had a > 15% change in access resistance were discarded. Recordings were analyzed with Clampfit 10.3.

RNA extraction and cDNA library preparation

Five P7 mice were decapitated in two different days/batches (three mice in batch 1 and two in batch 2), the brains extracted and sectioned at a vibratome in ice-cold extracellular solution (see *In vitro electrophysiology*). Sensory (SZ, *stratum zonale*; SGS, *stratum griseum superficiale;* SO, *stratum opticum)* and motor (SGI, *stratum griseum intermedium;* SAI, *stratum album intermedium;* SGP, *stratum griseum profundum;* SAP, *stratum album profundum*) domains were manually dissected from the slices and the tissue processed for total RNA extraction using an RNeasy Mini kit (QIAGEN, Venlo, Netherlands). Four samples were obtained from each mouse: sensory anterior SC, sensory posterior SC, motor anterior SC and motor posterior SC. cDNA libraries were generated from total RNA using a TruSeq® Stranded Total RNA Library Prep Kit (Illumina, San Diego, CA) and sequenced on an Illumina HiSeq 4000 machine (single read, 50bp read length).

Immunohistochemistry

For immunohistochemistry (IHC) experiments, mice were anaesthetized with Euthatal (0.2 ml) and perfused with 20 mL of ice cold phosphate buffered saline (PBS) followed by 20 mL of 4% paraformaldehyde (PFA) in PBS. Brains were incubated in PFA overnight at 4°C and then dehydrated for 48 hours in 30% sucrose in PBS at 4°C. The brains were frozen in O.C.T. compound (VWR, Radnor, PA) and sliced into 40 μm sections using a CM1950 cryostat (Leica, Wetzlar, Germany).

Free-floating sections were rinsed in PBS and incubated in blocking solution (1% bovine serum albumin and 0.3% Triton X-100 in PBS) containing primary antibodies for 24 hours at 4°C. Sections were washed with PBS four times at room temperature and incubated for 24 hours at 4°C in blocking solution with secondary antibodies. Immuno-labeled sections were washed four times with PBS at room temperature (RT) and mounted on glass slides (SuperFrost Plus, Thermo Scientific, Waltham, MA) using DAPI Fluoromount-G® (SouthernBiotech, Birmingham, AL). Primary antibodies used in this study were: chicken anti-GFP (Aves Labs, GFP-1020, 1:2000), rabbit anti-RFP (Rockland, 600-401-379, 1:2000), mouse anti-NeuN (Millipore, MAB377, 1:1000) and chicken anti-β-Galactosidase (Abcam, ab9361, 1:2000). Secondary antibodies used were Alexa Fluor 488 donkey anti-chicken (Jackson ImmunoResearch, 703-545-155, 1:1000), Cy3 donkey anti-rabbit (Jackson ImmunoResearch, 711-165-152, 1:1000) and Cy5 donkey anti-mouse (Jackson ImmunoResearch, 715-175-150, 1:1000). Images were acquired using a Zeiss780 confocal microscope using a 20x/0.8NA air lens (Carl Zeiss AG, Jena, Germany).

In situ hybridization

Pitx2-CRE::Rosa-LSL-tdTomato mice were used in these experiments; brains were extracted, fresh frozen in O.C.T. and sliced into 15 μm sections at the cryostat. Brain sections were mounted on glass slides immediately after cryosectioning using an anti-roll blade and stored at −80°C for up to two weeks. On the day of the assay, sections were post-fixed for 30 minutes with ice-cold 4% PFA in PBS and then rinsed twice in PBS. Tissue pre-treatment and probe hybridization were carried out using RNAscope® reagents and experimental protocols (Bio-Techne, Minneapolis, MN). Briefly, Protease IV was applied on the slides for 20 minutes at RT and the probes were warmed for 10 minutes at 40°C to ensure that the reagents were homogenized. Slides were rinsed twice in PBS and the target probes added to the slices and allowed to hybridize for 2 hours. The slides were rinsed twice in wash buffer and then underwent a series of hybridization steps through which fluorescent signals were assigned to a specific probe channel (Amp 1-FL for 30 minutes, Amp 2-FL for 15 minutes, Amp 3-FL for 30 minutes, Amp 4-FL-Alt C for 15 minutes). All hybridization steps were performed in the HybEZ® oven at 40°C and were separated by washes in wash buffer. Slices were counterstained with DAPI for 30 s at RT and cover-slipped with ProLong Gold Antifade mountant (Thermo Fisher Scientific, Waltham, MA). On the following day, sections were imaged using a Zeiss780 confocal microscope using a 63x/1.4NA oil lens (Carl Zeiss AG, Jena, Germany). The probes used in this study were: anti-vGluT2 (Mm-Slc17a6-C2, 319171-C2), anti-vGAT (Mm-Slc32a1, 319191), anti-tdTomato (tdTomato-C3, 317041-C3) and anti-Pitx2 (Mm-Pitx2-C2, 412841-C2).

Whole-brain clarification and imaging

Pitx2-CRE::Rosa-LSL-tdTomato mice were perfused and the brains fixed overnight as described in *Immunohistochemistry.* Brain clarification was carried out following the CUBIC protocol [76]. Briefly, the brains were immersed in 0.5x reagent-1A (10wt% Triton, 5 wt% Quadrol, 10 wt% Urea, 25 mM NaCl in dH_2O, diluted in dH_2O) for 6 hours at RT and then 1x reagent-1A for two days at RT with shaking. Fresh reagent-1A was then replaced and the samples placed at 37°C with shaking; reagent-1A was replaced every other day for a total of 10 days of incubation. The brains were then washed in PBS at RT and incubated with 0.5x reagent-2 (25 wt% urea, 50 wt% sucrose, 10 wt% triethanolamine in dH_2O, diluted in PBS) for 24 hours at RT, followed by an incubation in 1x reagent-2 for 2 days at RT. Brains were allowed to equilibrate in a 50:50 silicon oil/mineral oil mix, then imaged in the same medium using an UltraMicroscope II light sheet microscope (LaVision Biotech, Bielefeld, Germany).

Surgical procedures

All procedures using live animals were approved by the Home Office and the LMB Biosafety committee. Mice were anaesthetized with isoflurane delivered at a flow of 3% in 2 L/min of O_2 for the initial induction and then maintained at 1%–2% in 2 L/min of O_2. The anaesthetized animal was placed into a stereotaxic apparatus (David Kopf Instruments, Tujunga, CA) and Rimadyl (2 mg/kg body weight) was administered subcutaneously as anti-inflammatory. Both eyes were covered with Lubrithal eye gel (Dechra Pharmaceuticals, Northwich, UK) to prevent corneal desiccation during the surgery and the body temperature was maintained at 37°C through a CMA 450 Temperature Controller (Linton Instrumentation, Norfolk, UK) for all the surgical procedure. After skin removal, two sterile screws were inserted in the in the frontal part of the skull to give more stability to the implant. A craniotomy (1 mm diameter) was drilled to expose the area of interest and, after removal of the dura, we performed one of the following procedures: viral injection, optic fiber implantation, optetrode implantation or multi-fiber array implantation. For head restriction experiments, a head-plate was glued to the skull before drilling the craniotomy. For multi-fiber array implantation, a larger craniotomy (1.5 mm diameter) was drilled. The remaining exposed brain was then covered with sterile vaseline and the implant was sealed with RelyX^{TM} Unicem2 Self Adhesive Resin Cement (3M, Bracknell, UK). All mice were monitored after surgery and given at least one week to recover before recording.

Viral injections

Viruses were injected using a 5 μl Hamilton syringe (Scientific Laboratory Supplies, Nottingham, UK) equipped with a 33G needle. The syringe was left in the brain for 5 min before being retracted. Up to a maximum of 500 nL of virus were injected in the SC (coordinates: $x = -0.80$, $y = -3.80$, $z = 1.70$). For anterograde tracing, the following viruses were co-injected: AAV(1)-CMV-FRT-TVAmCherry-2A-Gly (titer: $3.9x10^{12}$ genomic copies/ml) and AAV(9)-CMV-FRT-SynGFP-WPRE (titer: $3.0x10^{12}$ genomic copies/ml). For retrograde tracing, AAV(1)-CMV-FRT-TVAmCherry-2A-Gly (titer: $3.9x10^{12}$ genomic copies/ml) was injected at day 0, followed by injection of virus EnvA-ΔG-Rabies^{GFP} (titer: $4.3x10^{8}$ infectious units/ml) at day 21 through the same craniotomy. Mice were perfused three weeks after viral injections of AAV constructs for anterograde tracing or one week after injection of Rabies virus for retrograde tracing. Brain tissue was processed as described in *Immunohistochemistry*.

3D tracking of head-over-body position

We used sensor boards [23] to track the position of freely moving animals in an open field Perspex arena (50x50cm). In order to obtain the orientation of the mouse head relative to the body, we secured two inertial sensors on the head and the body of the animal using Mill-Max connectors and an elastic band respectively. The orientation of the head relative to the body was calculated as the difference between the head sensor and the body sensor outputs. All sensor data were acquired at 50 Hz. Prior to any recording session, we verified sensor alignment and recalibrated the boards if necessary as previously described [23]. The validity of the inertial sensor system for outputting reliable positional information was tested statically and while undergoing rotations at a range of velocities. To test for drift in the static regime, each sensor was fixed to a base plate in the recording arena and recorded for 30 minutes. Drift in the system was tested by determining the shift in the sensor output between each measurement (jitter) and the cumulative change in heading over the course of the 30 minutes recording (cumulative drift). To test their ability to track motion occurring at different velocities, the sensors were fixed to step-motor controlled rotation table and their recordings compared to the expected motion pattern applied. The table was set to rotate at four speeds (28°/s, 40°/s, 56°/s and 80°/s), applied to both clockwise and counterclockwise directions. Recordings were carried out ten times for each direction and velocity. The expected angular displacement between each 50 Hz measurement (28°/s = 0.56°, 40°/s = 0.8, 56°/s = 1.12° and 80°/s = 1.6°) was then compared with the computed displacements between each temporal bin from the sensor output. The measurement of error was then transformed to give a measurement error per degree for each temporal bin.

Optogenetic stimulation in freely moving animals

For optogenetic manipulation experiments, *Pitx2-Cre::Rosa-LSL-ChR2-eYFP* mice were implanted with single optic fibers or custom-made optic fiber arrays (core = 100 μm, NA = 0.22; Doric Lenses, Québec, Canada) in the SGI. Fiber array design is presented in Figure S4K. Implant coordinates for single fibers are presented in Figure S6E; implant coordinates from bregma for optic fiber arrays were: $x = -3.7$ mm, $y = 0.8$ mm and $z = 1.9$ mm (from tip of fiber 1). WT mice were implanted as controls.

Light for optical stimulation was delivered by a 473 nm laser diode module coupled to a 100 μm multimode fiber (NA = 0.22) through a Schäfter + Kirchhoff fiber coupler (Cobolt, Solna, Sweden). For single-site stimulation experiments, light was delivered to the implanted optic fiber through a fiber-optic patchcord (core = 100 μm, NA = 0.22). For multi-fiber array stimulation, we used a custom-made optical fiber branching patchcord (core = 50 μm, NA = 0.22). All fiberoptic patchcords were from Doric Lenses, Québec, Canada. The laser power employed in all stimulation experiments was 3-5 mV at the tip of the fiber. All optogenetic stimulation experiments were carried out in an open field arena; mice were acclimatised to the set up for 10 minutes before starting the recording sessions. Each recording session consisted of an initial 10 s during which no light stimulation was provided, followed by 10 repetitions of a 250 ms continuous light pulse every 10 s. In the frequency modulation assay, the stimulus consisted of a train of 10 ms pulses presented at 10, 20, 30, 40 or 50 Hz lasting for 250 ms. In the duration modulation assay, the stimulus consisted of a continuous light pulse of 250, 500, 1000 or 2000 ms. For both assays, each recording session contained only one stimulation type presented 10 times. Each animal underwent at least 3 recording sessions per stimulation type. For histological confirmation of implant position, mice were perfused and their brain tissue processed and sectioned as described in *Immunohistochemistry*. Images of implant locations were referenced against the Allen Mouse Brain Atlas to estimate implant coordinates.

Optogenetic stimulation in head-restrained animals

For eye tracking experiments, *Pitx2-Cre::Rosa-LSL-ChR2-YFP* mice were implanted with two optic fibers (core = 100 μm, NA = 0.22; Doric Lenses, Québec, Canada), one in each hemisphere in the SGI, and a head plate. WT mice were implanted as controls. Light stimulation was provided by a solid-state 473 nm laser (Ikecool Coorporation, Los Angeles, CA). Awake mice were head-fixed and allowed to run on a wheel in dark conditions while their eyes were recorded using a DMK 23UM021 camera (Imaging Source, Bremen, Germany) equipped with MVL7000 lens with infrared illumination provided by a 850 nm Infrared LED array light source (Thorlabs, Newton, NJ). The camera was pointed at the center of the eye, as gauged from the resting position of the pupil. Each recording session lasted 5 minutes; light pulses lasting 250 or 500 ms were repeatedly delivered every 10 s for the entire duration of the session for a total of 30 repetitions of light stimulation per session. Each animal underwent at least 2 recording sessions per stimulation type.

In vivo electrophysiology

Pitx2-CRE::Rosa-LSL-ChR2-eYFP mice were implanted with moveable 17 μm-diameter platinum-iridium (H-ML insulated) microelectrodes (California Fine Wire, US), configured as four tetrodes carried by 16 channel microdrives (Axona Ltd., St. Albans, UK). Tetrodes were platinum electroplated to an impedance of 100-250 kOhm using a Kohlraush/Gelatin (9:1, 0.5% gelatin) solution. In order to opto-tag Pitx2-ChR2 expressing neurons, tetrodes were combined with an optic fiber (core = 100 μm, NA = 0.22; Doric Lenses, Québec, Canada). The resulting optetrodes were implanted just dorsally to the intermediate layers of the superior colliculus at co-ordinates 3.8-4.2 mm posterior from Bregma, 1.25 mm lateral of the midline and 1.3-1.5 mm ventral to the brain surface. Single-units were recorded as mice foraged a square Perspex arena (50 × 50 cm) for droplets of 30% diluted soy milk. Recording sessions consisted of ten five-minute epochs, six of which performed with different protocols of blue light stimulation (Figure S4M). The remaining four epochs were carried out without blue light stimulation, with the first and last epochs occurring in light condition and the second and third in darkness. The recording arena was situated within a Faraday cage containing stable polarizing cues. Light trials were recorded with one door of the Faraday cage open, while the arena was completely enclosed during dark trials. During dark trials all other sources of light within the experimental room such as computer screens were switched off or covered. Recordings were carried out using a multi-channel DacqUSB recording system (Axona Ltd., St Albans, UK). In order to record units, animals were connected to a pre-amplifier via a lightweight cable attached to the microdrive by a head-stage that modified the signal with AC-coupled, unity gain operational amplifiers. The signal was amplified ∼12-20000 times and bandpass filtered between 500 Hz and 7 kHz. Recording thresholds were set to ∼70% above baseline activity levels, and data from spikes above the threshold from all channels were collected across a period spanning 200 μs preceding and 800 μs following the peak amplitude of a spike. The activity of channels from any given tetrode was referenced against the activity of a single channel from another tetrode, so as to increase the signal to noise ratio. Tetrodes were advanced ventrally into the brain by 50 μm after each day of recording.The optic fiber was coupled with a blue diode pumped solid-state 473 nm laser (Ikecool Coorporation, Los Angeles, CA) pulsed at 30 Hz with 5 ms pulse width and a 3-5 mW at the tip of the fiber. Blue light-activated units were defined on the basis of the latency of the response to a pulse of light within a time window of 5 ms [77]. The inertial sensor was attached to the head-stage on the head of the mice using Mill-Max connectors. The signal from the sensor was passed through a lightweight cable via one Arduino for processing the signal and computing the DCM algorithm and a second for controlling synchronization with the DacqUSB single unit recording system. The control Arduino was connected to the DacqUSB system using the system's Digital I/O port. A custom built BASIC script was written in DacqUSB to synchronize the start of single-unit recording and the start of the blue laser with the key-press initiation of inertial sensor recording (controlled using the Processing software sketchbook; processing.org).

QUANTIFICATION AND STATISTICAL ANALYSIS

In vitro electrophysiology

The neuronal classification was obtained using a K++ hierarchical clustering algorithm [30] as implemented in the Orange© Data Mining Library available for Python. For each recorded neuron the following electrophysiological properties were used to compute the hierarchical clustering (dendrogram) and the distance map: passive membrane properties; access resistance (Racc, MOhm), resting membrane potential (RMP, mV), input resistance (Rinput, MOhm), time constant (τ, ms), capacitance (Cp, pF); action potential properties; threshold (mV), amplitude (mV), width (ms), after-hyperpolarization amplitude (AHPamp, mV) and width (AHPwidth, ms); firing profile (from 20 to 400 pA injected); frequency (Hz), frequency of adaptation (%), frequency between the first and third action potential (Hz), delay of the first action potential (ms), delay of the last action potential (ms).

RNA-seq data processing

Reads were trimmed using Trim Galore (version 0.4.2) with default parameters to remove the standard Illumina adaptor sequence. Reads were mapped to the GRCm38 assembly using STAR version STAR_2.5.3a_modified [74]. Raw read counts per transcript were calculated using HTSeq version 0.9.1 on GRCm38.p5 gene set using directional counts. Differential analysis of gene expression was performed using the default settings in DESeq2 [75]. Given that replicates were produced in two different batches, the statistical model was designed to take into account batch effects in the data by including two factors in the design formula: region in the SC (superficial versus deep) and batch (batch1 versus batch2). Differentially expressed genes were called at padj < 0.05. Replicate SP_1 for the sensory posterior SC was classified as an outlier and excluded from the analysis.

Histological analysis

For quantification of the relative abundance of Pitx2^{ON} as a fraction of total neuronal population (marked by expression of NeuN) in the SGI, we sampled 9 coronal sections across different AP coordinates and counted the proportion of Pitx2^{ON}/NeuN only cells in the medial (x: 0-0.5), medial/lateral (x: 0.5-1.0) and lateral (x: 1.0-1.4) SGI. For quantification of Pitx2^{ON} neurons distribution across the brain, we counted Pitx2^{ON} neurons in all thin sections obtained from three *Pitx2-CRE::Rosa-LSL-tdTomato* mice. For quantification of the co-localization of *Pitx2*-driven tdTomato with glutamatergic and GABAergic markers, we sampled 11 coronal sections across different AP coordinates. For quantification of the co-localization of *Pitx2*-driven tdTomato with *Pitx2*, we sampled 3 coronal sections for each developmental stage (P7 and P60). For tracing experiments, images of mounted sections were automatically detected and acquired using a robot-assisted Nikon High Content Analysis microscope equipped with a 10x air objective (0.45 NA) operated by NIS-Elements HC software (Nikon, Tokyo, Japan). The assignment of both input and output signals to specific brain areas was carried out manually aligning the acquired images to the Allen Mouse Brain Atlas. For quantification of synaptic density in anterograde tracing experiments, the average pixel intensity in a target region containing SynGFP punctae was calculated in ImageJ (NIH, Bethesda, MD) and the background was subtracted using an adjacent unlabelled region as reference.

Light sheet microscopy imaging analysis

Quantification of Pitx2-defined SGI periodicity was analyzed by cropping a spherical cap in the 3D brain image using the G'MIC (gmic.eu) image processing suite. Projected volumes were further filtered using a Difference of Gaussian filter and a segmentation was obtained by thresholding the response of the filter using a script in MATLAB (MathWorks, Natick, MA).

Characterization of 3D head movements

Head motion events for each Eulerian component were defined as events in which the angular head velocity remained in a constant direction for at least five temporal bins (a total of 100 ms) at a speed of over 0.5° per bin (25°/s). This definition was further refined by searching backward from the onset of the initially defined motion to the last temporal bin at which direction was the same as the defined motion; this was now defined as the onset of motion. A similar process was also carried out to define the offset of motion, the last temporal bin from the initially defined offset of motion to have the same direction as the defined motion was considered as the final offset of motion. From these values we retrieved the total extent of motion (the summation of the angular head velocity for a motion event) and the duration of motion for each motion event. This process was carried out separately for each Eulerian component. Frequency histograms were created for the head displacements for each animal, taken from the light trial recordings. The computed head displacements were grouped into 10° bins and normalized based on the maximum sampling frequency for the creation of the frequency histograms. Gaussian curves were fit to the resulting distributions and the peak, mean and sigma of the fit were retrieved from the fitted model.

In vivo optogenetics

Successful events were defined as episodes in which a motion event (see definition in *Analysis of head motion events*) was detected within 2 s of light onset. Success rate for a given stimulation type was calculated as average success rate among sessions in which the same stimulation parameters were applied. The extent of the light-triggered motion was calculated as difference between the maximum head-over-body displacement observed within 2 s of light onset and the baseline head-over-body position (calculated as average position in the 1 s preceding light onset). Mean light-triggered head displacement was determined averaging successful events only. Individual trials of optogenetic stimulation were baseline-subtracted and analyzed for stepwise movements as follows. The velocity of head displacements was calculated as the derivative of the head position. We defined as movement onsets moments when the head velocity exceeded 25°/s. This threshold was chosen in accordance with our definition of movement (see *Characterization of 3D head movements*). The termination of a movement was defined as the instant when the head velocity either inverted direction or reached a minimum between consecutive steps, or when the head position reached the maximum displacement attained during the trial. The amplitude of each motion step was defined as the difference between the head position at movement termination and the position at movement onset. For every light stimulation used, we calculated the number of steps per trial, the fraction of trial presenting a given number of steps for progressively longer stimulation and the average step amplitude, duration and velocity. In order to estimate the magnification factor for motor representation in the SC, the average maps of yaw, pitch and roll head rotation were smoothed with a 250 μm wide Gaussian kernel. The gradient direction at each location on the array was calculated as:

$$\nabla \boldsymbol{A} = \frac{\partial \boldsymbol{A}}{\partial x} + \frac{\partial \boldsymbol{A}}{\partial y}$$

where A correspond to the map of yaw, pitch or roll, and x and y represent spatial coordinates on the array. The map of magnification factor (mm^2/deg^2) [43], was estimated using the inverse of the Jacobian determinant computed from the yaw and pitch gradients at each location of the array (distance between fibers = 500 μm):

$$Magnification = \begin{vmatrix} \partial \boldsymbol{Y}/\partial x & \partial \boldsymbol{Y}/\partial y \\ \partial \boldsymbol{P}/\partial x & \partial \boldsymbol{P}/\partial y \end{vmatrix}^{-1}$$

Eye tracking

Eye tracking videos were processed offline using a custom-made software in MATLAB (Mathworks, Natick, MA). Briefly, each frame was low-pass filtered to reduce noise and the pupil outline was detected by a level-crossing edge detector; the position and the area of the pupil were calculated from the ellipse fit to the pupil outline. The output of the algorithm was visually inspected and adjustments to the parameters (e.g., spatial filter strength, or level-crossing threshold) were made where needed. The angular amplitude of eye movements relative to the resting position was estimated as: $\alpha = \arcsin(d/r)$, where d is the distance traveled by the pupil center and r is the radius of the eye, approximated to a sphere. Eye light triggered average (LTA), movement amplitude and stepwise movement analysis were carried out as for head movements.

Spike sorting

The electrophysiological data were spike sorted using TINT cluster cutting software (Axona, St Albans, UK). Cluster cutting was carried out by hand as clusters were generally well separated. Clusters were included in analysis if they exhibited over 100 spikes for each trial recording sessions and did not belong to clusters identified in previous recording trials. The same isolated and responsive units were recorded across multiple epochs (Figures S4L and S4M).

Determination of motion tuning

The motion tuning of SC neurons was determined by carrying out spike triggered averages (STA) of head displacements. In order to do this, we first defined spike events, aligned corresponding head displacements to these events and compared the computed average displacement vectors with those drawn from a random distribution. Cells were only considered to be tuned to a given component if the motion vector of the cell for that component was above threshold and in the same direction in both light trials.

Spike and light triggered average

For the STA of motion the angular head velocity for the 50 (1 s) temporal bins preceding and 100 bins (2 s) following the onset of spike were computed for each Eulerian component. The direction of the head at the onset of each spike onset was normalized to zero for each Eulerian component. The calculated angular head velocities (AHV) were cumulatively summated for each temporal bin to produce a head displacement for the 1 s preceding and 2 s following the onset of spike. This was repeated for each spike. The mean and SEM of spike related head displacements were then calculated for each temporal bin to illustrate the tuning of neurons. Displacement vectors were calculated as the difference between the minimum and maximum of the computed displacement for each spike event, and the mean displacement vector for each Eulerian component was computed from each neuron's computed displacement vectors for the given component. The direction of the displacement vector was defined according to the temporal order of the minimum and maximum values of the computed displacement (i.e., if the minimum preceded the maximum value, the displacement was deemed to be positive). For LTA the time of light onset was used in place of the spike onset.

Generation of shuffled datasets

For each cell, the spike-onset times were temporally shifted by 20-150 s (selected from a random distribution) in a wrap-around manner. This works to shift the relationship between the bursting times and the recorded heading directions of the animals while maintaining the temporal relationship between bursting events. Once these data were shifted, spike triggered analyses were carried out to determine the mean displacement vector of the temporally-shifted data. This process was repeated 1000 times so as to produce a random distribution of mean displacement vectors.

Statistical methods

Data were tested for normality before statistical analysis. For measurements of sensor boards' error during motion, errors per temporal bin were compared using a two-way 4x2 factorial ANOVA. For the assessment of movement range in freely moving animals in our experimental setup, repeated-measures ANOVA was used to compare standard deviations of the fit curves. For the analysis of stepwise kinematics, one-way ANOVA was used to compare amplitude, duration and velocity of individual steps within full motion bouts. To assess the effect of stimulus duration on the head and eye movements, paired t tests were used to compare amplitude of head and eye displacements following 250 and 500 ms light pulses. For optogenetic stimulation experiments with multi-fiber arrays, one-way ANOVA and Tukey multiple comparison tests were used to assess the difference between the amplitude of displacement generated at various SC locations. For *in vivo* recordings of Pitx2^{ON} units, paired t tests were used to assess the stability of tuning curves under different light conditions. In all STA and LTA analysis, a t test was applied between the displacement vector and the mean displacement vector of the shuffled data with a threshold of 0.05. Neurons were considered to be motion tuned if the t test determined a significant difference between the displacement vectors of the real and shuffled data.

All results are presented as mean ± SEM. Results were considered statistically significant at $^{*}p \leq 0.05$, $^{**}p \leq 0.01$, $^{***}p \leq 0.001$. Relevant *p-value*s are reported in the figure legends.

DATA AND CODE AVAILABILITY

Data analysis in this paper was performed using MATLAB (R2014b), Python 2.7, GraphPad Prism7 and R (version 3.5.0). The data and code that support the findings of this study are stored on the LMB server and are available from the corresponding author upon reasonable request. The accession number for the deep-sequencing gene expression data reported in this paper in Gene Expression Omnibus (GEO) is: GSE135082.

Current Biology, Volume *29*

Supplemental Information

Genetically Defined Functional Modules for Spatial Orienting in the Mouse Superior Colliculus

Laura Masullo, Letizia Mariotti, Nicolas Alexandre, Paula Freire-Pritchett, Jerome Boulanger, and Marco Tripodi

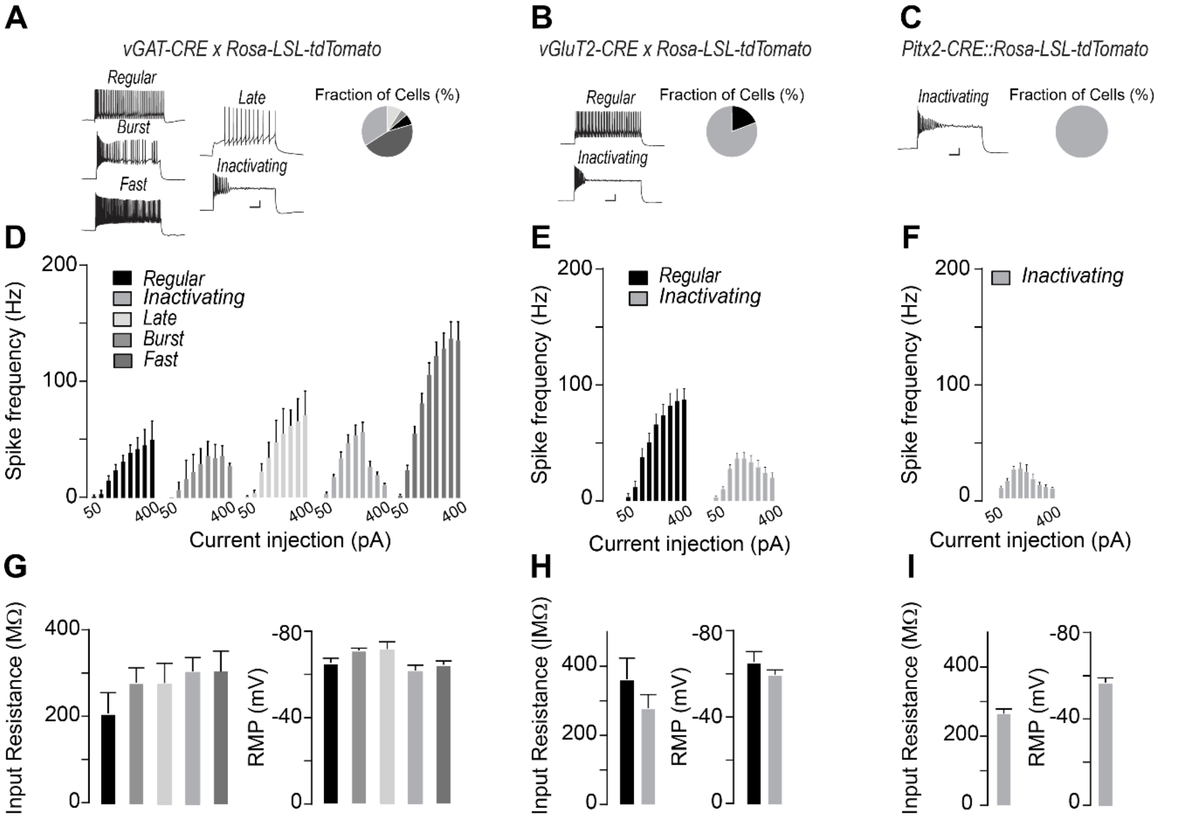

Figure S1 │ *In vitro* characterization of vGAT^{ON}, vGluT2^{ON}, and Pitx2^{ON} neurons in the SC motor domain. Related to Figure 1.

A-C, Representative firing profiles of vGAT^{ON} (**A**), vGluT2^{ON} (**B**), and Pitx2^{ON} (**C**) neurons (scale bars: 20 mV, 100 ms; 200 pA of current injected). Inset: fraction of cells per firing profile. **D-F**, Membrane potential response to steps of increasing positive current (50-400 pA) in vGAT^{ON} ($n_{NEURONS}$ = 46, n_{MICE} = 17) (**D**), vGluT2^{ON} ($n_{NEURONS}$ = 26, n_{MICE} = 5) (**E**), and Pitx2^{ON} (n_{NEURONS} = 16, n_{MICE} = 5) (**F**) neurons clustered according to their firing profile. **G-I**, Input resistance and resting membrane potential (RMP) of vGAT^{ON} (**G**), vGlut2^{ON} (**H**) and Pitx2^{ON} (**I**) neurons. All results are presented as mean ± SEM.

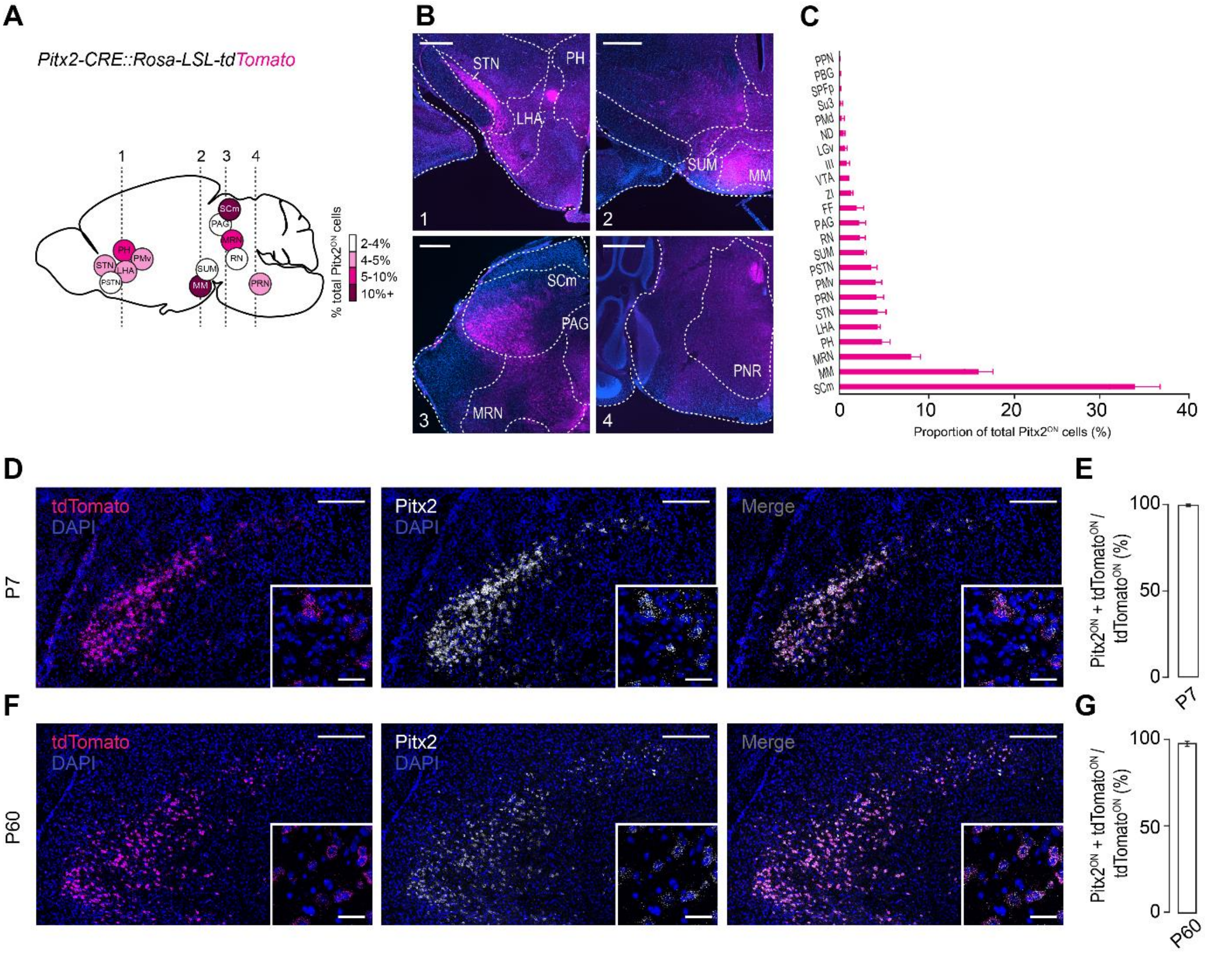

Figure S2 │ Pitx2 expression in mouse brain. Related to Figure 1.

A, Schematic representation of the top 12 brain regions containing Pitx2^{ON} cells, studied in a *Pitx2-CRE::Rosa-LSL-tdTomato* mouse ($n_{ANIMALS}$ = 3). **B**, Representative histologies highlighting presence of Pitx2^{ON} cells in coronal sections marked with dashed lines in **A** (scale bars: 500 µm). **C**, Quantification of distribution of Pitx2^{ON} cells across the brain, studied in a *Pitx2-CRE::Rosa-LSL-tdTomato* mouse ($n_{ANIMALS}$ = 3). **D-G**, Representative histologies and quantification of double *in situ* hybridization in *Pitx2Cre::Rosa-LSL-tdTomato* mice at P7 (**D, E**) and P60 (**F, G**), with *tdTomato* and *Pitx2* probes (scale bar: 100 µm, insets: 30 µm; n_{SLICES} = 6). All results are presented as mean ± SEM.

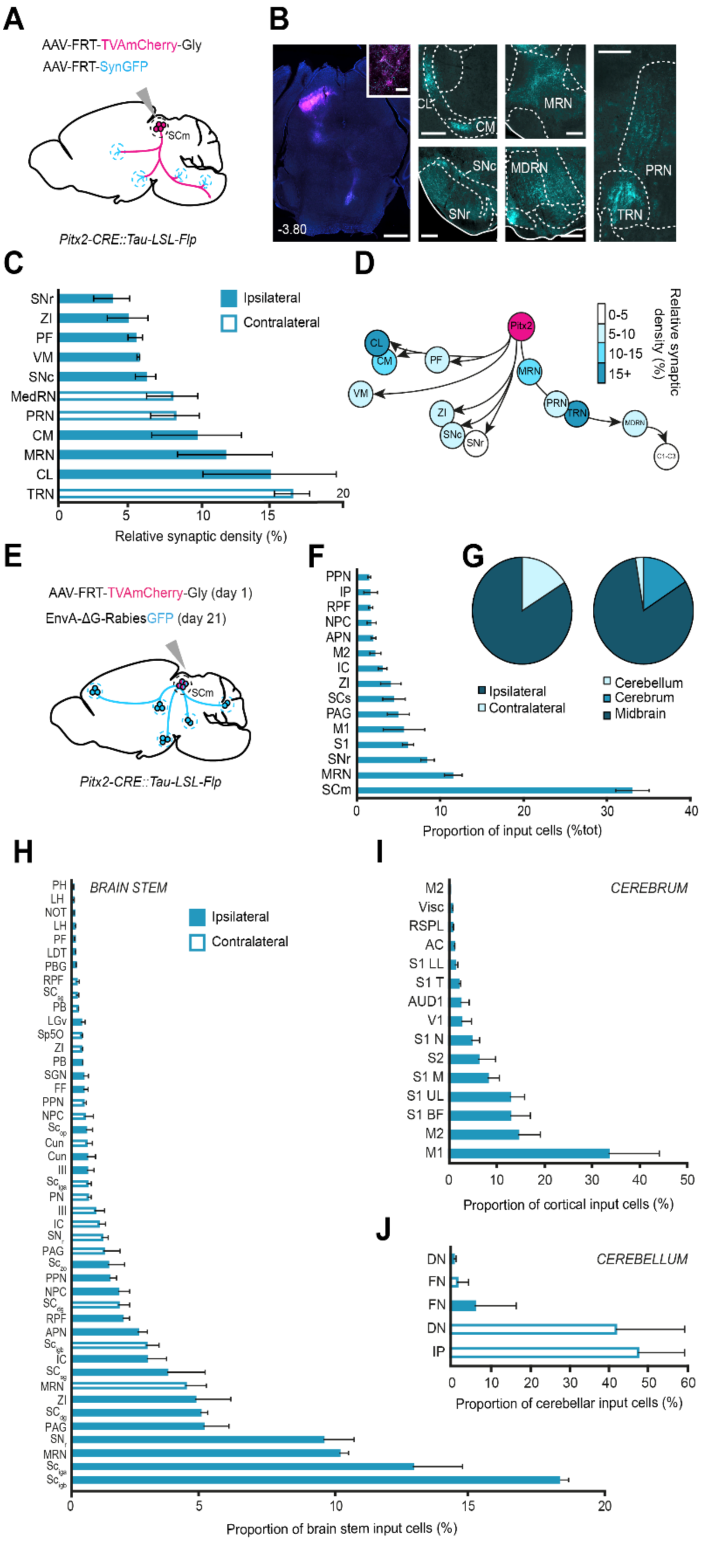
A
AAV-FRT-TVAmCherry-Gly
AAV-FRT-SynGFP
SCm
Pitx2-CRE::Tau-LSL-Flp
B
-3.80
CL
CM
MRN
SNc
SNr
MDRN
PRN
TRN
C
Ipsilateral
Contralateral
SNr
ZI
PF
VM
SNc
MedRN
PRN
CM
MRN
CL
TRN
0
5
10
15
20
Relative synaptic density (%)
D
Pitx2
CL
CM
PF
VM
MRN
PRN
TRN
MDRN
ZI
SNc
SNr
0-5
5-10
10-15
15+
Relative synaptic density (%)
E
AAV-FRT-TVAmCherry-Gly (day 1)
EnvA-ΔG-RabiesGFP (day 21)
SCm
Pitx2-CRE::Tau-LSL-Flp
F
PPN
IP
RPF
NPC
APN
M2
IC
ZI
SCs
PAG
M1
S1
SNr
MRN
SCm
0
10
20
30
40
Proportion of input cells (%tot)
G
Ipsilateral
Contralateral
Cerebellum
Cerebrum
Midbrain
H
BRAIN STEM
Ipsilateral
Contralateral
PH
LH
NOT
LH
PF
LDT
PBG
RPF
PB
LGv
Sp5O
ZI
PB
SGN
FF
PPN
NPC
Cun
Cun
III
PN
III
IC
PAG
PPN
NPC
RPF
APN
IC
MRN
ZI
PAG
MRN
0
5
10
15
20
Proportion of brain stem input cells (%)
I
CEREBRUM
M2
Visc
RSPL
AC
S1 LL
S1 T
AUD1
V1
S1 N
S2
S1 M
S1 UL
S1 BF
M2
M1
0
10
20
30
40
50
Proportion of cortical input cells (%)
J
CEREBELLUM
DN
FN
FN
DN
IP
0
10
20
30
40
50
60
Proportion of cerebellar input cells (%)

Figure S3 │ Viral tracing of pre- and post-synaptic networks of the Pitx2^{ON} population. Related to Figures 2 and 7.

A, Anterograde tracing of Pitx2^{ON} neurons projections in *Pitx2-CRE::Tau-LSL-FLPo-INLA* mice targeted via FLP-dependent AAVs expressing synaptic (*cyan*, SynGFP) and membrane (*magenta*, TVAmCherry) markers (n_{MICE} = 3). **B**, Overview of infected neurons (scale bar: 1 mm, inset: 50 µm) and their extra-collicular targets (scale bars: 300 µm). **C-D**, Schematic representation (**C**) and quantification (**D**) of relative synaptic density of Pitx2^{ON} neurons terminals in target areas (Central lateral nucleus of the thalamus, CL; Central medial nucleus of the thalamus, CM; Ventral medial nucleus of the thalamus, VM; Parafascicular nucleus, PF; Zona incerta, ZI; Substantia nigra pars compacta, SNc; Substantia nigra pars reticulata, SNr; Midbrain reticular nucleus, MRN; Pontine reticular nucleus, PRN; Tegmental reticular nucleus, TRN; Medullary reticular nucleus, MDRN; Spinal cord, cervical segments C1-C3; n_{MICE} = 3). **E**, **F**, Schematic representation (**E**) and quantification (**F**) of top 15 input regions to Pitx2^{ON} neurons, studied in *Pitx2-CRE::Tau-LSL-FLPo-INLA* mice via FLP-dependent AAV expressing TVA-Gly (*magenta*, TVAmCherry) followed by EnvA-ΔG-Rabies^{GFP} (*cyan*, GFP) injection (n_{MICE} = 3). **G**, Distribution of pre-synaptic partners of Pitx2^{ON} neurons in ipsi- and contralateral hemispheres to injection site (left) and across brain divisions (right). **H-J**, Distribution of input cells in cerebrum (**H**), cerebellum (**I**) and brain stem (**J**), expressed as percentage of total input cells in relative brain division (n_{MICE} = 3). All results are presented as mean ± SEM.

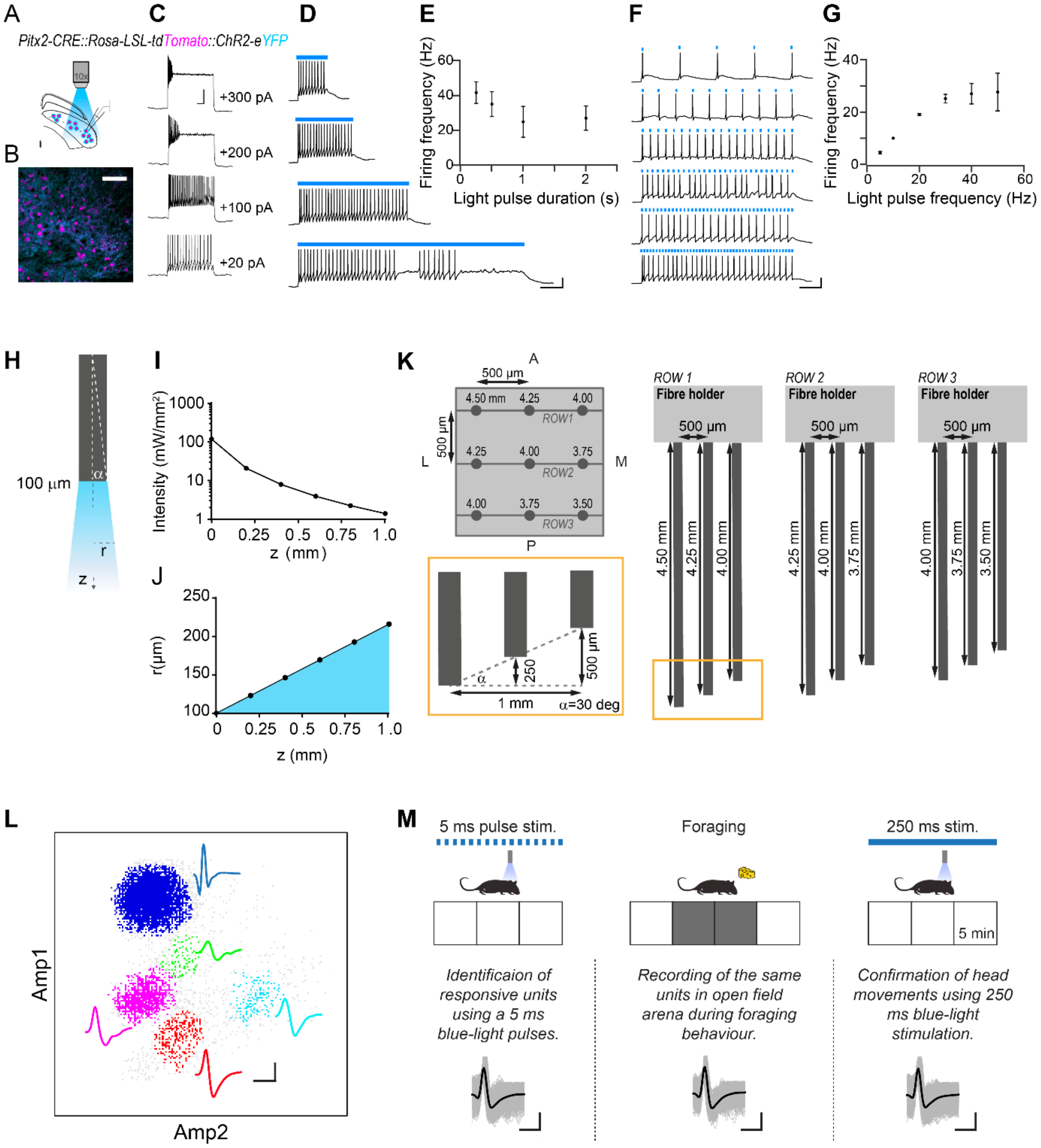

Figure S4 │ *In vitro* responses of ChR2 expressing Pitx2^{ON} neurons to various regimes of optical stimulation, specifics for single optic fibre and optic fibre array, and optotagging paradigm. Related to Figures 2, 4 and 6.

A, **B**, Schematic of recording setup (**A**) and visual targeting of Pitx2^{ON} neurons in slices from *Pitx2-CRE::Rosa-LSL-tdTomato::ChR2-eYFP* mice (**B**) (scale bar: 100 µm). **C**, Membrane potential response to steps of increasing positive current (20-300 pA current injected; scale bars: 100 ms, 20 pA). **D**, **E**, Membrane potential (**D**) and firing frequency (**E**) in response to blue light pulses of increasing duration. **F**, **G**, Membrane potential (**F**) and firing frequency (**G**) in response to 5 ms blue light pulses of increasing frequency (5-50 Hz) (scale bar: 20 ms, 20 pA; n_{NEURONS} = 7, n_{MICE} = 2). **H**, Schematic of light diffusion from optic fibre used for optogenetic stimulation *in vivo*. **I**, **J**,

Calculations of variation in light intensity (**I**) and radius (r) of light cone (**J**) as a function of the distance from fibre tip (z). **K**, Specifications of custom-made 3x3 optic fibre array and implant orientation with respect to mouse head; fibres are spaced at a distance of 500 µm along both the mediolateral and anteroposterior axes and fibre length is designed to accommodate for the spherical surface of the motor SC. All results are presented as mean ± SEM. **L**, Action potential amplitudes on two channels of a tetrode bundle showing five amplitude clusters (colour coded). Average action potential waveforms from the maximum amplitude channel are shown next to each cluster (scale bars: 50 µV, 2 ms). **M**, Experimental design: three epochs of 5 minutes each were recorded while the animal was free to explore an open field arena; in these epochs, 5 ms pulse stimulation at 30 Hz was used to isolate the responsive units. The same units were recorded during the following four epochs (two in light conditions, white blocks; and two in dark conditions, grey blocks) where the animal was exposed to soy milk drops. The last recording session consisted of three epochs during which 250 ms of continuous blue light stimulation was applied in order to confirm the characteristic vector for each animal recorded. Insets show the consistency of spike waveforms from a representative responsive unit across the three different recording sessions. Average action potential waveforms is in black, single action potential are in grey (scale bars: 50 µV, 2 ms).

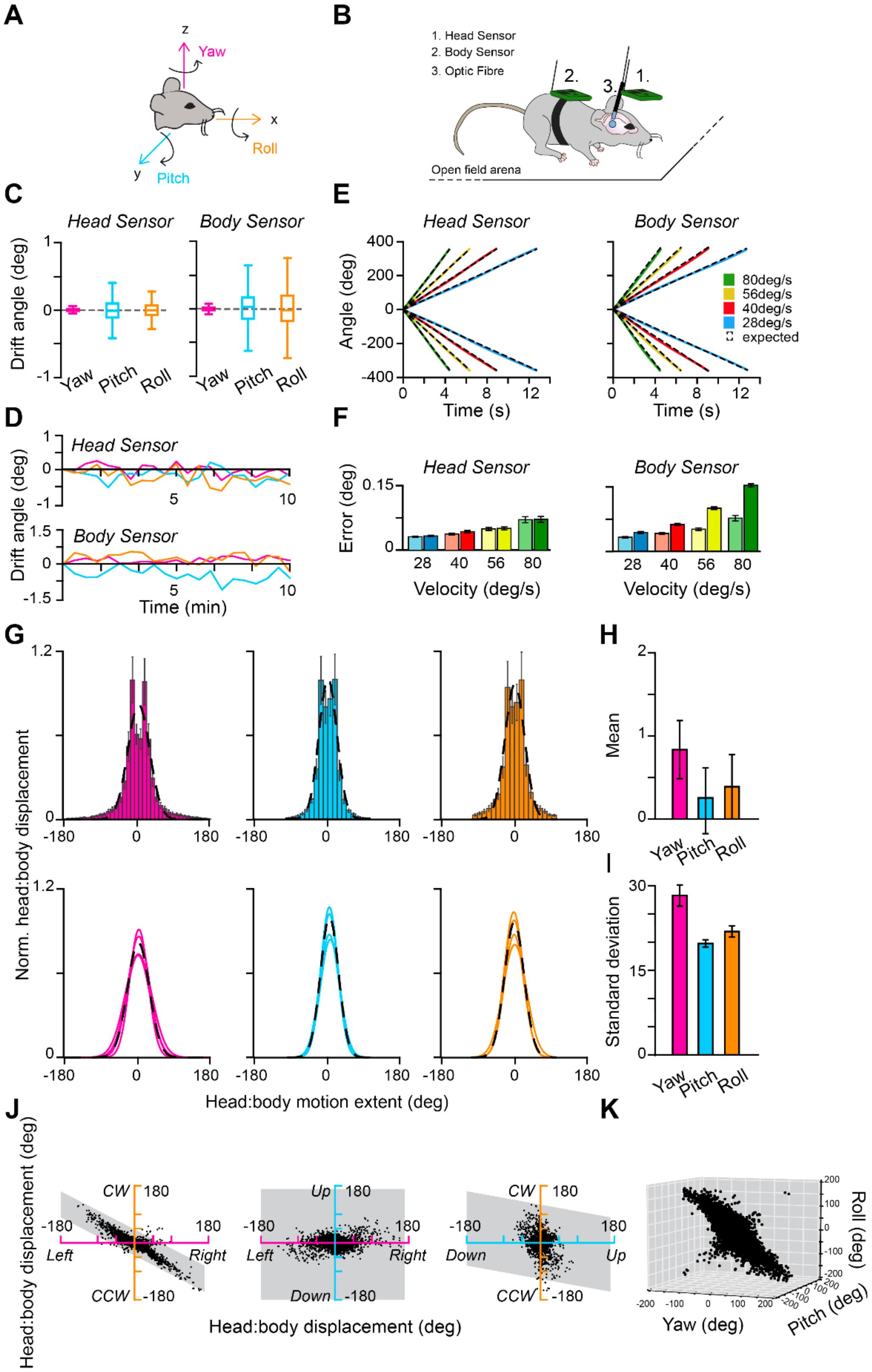
A
B
1. Head Sensor
2. Body Sensor
3. Optic Fibre
Open field arena
Yaw
Pitch
Roll
C
Head Sensor
Body Sensor
Drift angle (deg)
D
Time (min)
E
Angle (deg)
Time (s)
80deg/s
56deg/s
40deg/s
28deg/s
expected
F
Error (deg)
Velocity (deg/s)
G
H
Mean
I
Standard deviation
Norm. head:body displacement
Head:body motion extent (deg)
J
K
Head:body displacement (deg)
Left
Right
Up
Down
CW
CCW
Yaw (deg)
Pitch (deg)
Roll (deg)

Figure S5 │ Stability of double sensor readings and movement kinematics. Related to Figure 2.

A, Schematic of the colour-coded axes of rotation: yaw (*magenta*), pitch (*blue*) and roll (*orange*). **B**, Schematic of the *in vivo* optical stimulation recording setup with optic fibre and two sensor boards. **C**, Box and whisker plots showing the jitter in the sensors during static regime for each Eulerian dimension. **D**, Cumulative drift of the two sensors over 10 minute recordings in static regime. **E**, Sensors output recorded during step-motor-driven rotations over 360° at four different speeds and two directions (coloured lines) plotted against the expected trajectory (black dashed line). **F**, Bar plots showing the error for each sample at each speed (light shades for clockwise rotations and dark shades for counter-clockwise rotations; $p = 0.08$). **G**, Average head-over-body displacement for each axis of rotation recorded from freely moving animals, normalised to the maximum displacement produced ($n_{\mathrm{MICE}} = 4$) (top). Single animal average (solid lines) and population average (dashed line) of the Gaussian curve fitted to the histogram of head-over-body displacements ($n_{\mathrm{MICE}} = 4$) (bottom). **H**, **I**, Mean standard error (**H**) and mean standard deviation (**I**) of the Gaussian curves fitted in **G**. **J, K**, Eulerian representation of conjunctive motion from one representative mouse (**J**) and three-dimensional representation of yaw, pitch and roll displacements for all the mice (**K**) ($n_{\mathrm{MICE}} = 4$). All results are presented as mean ± SEM.

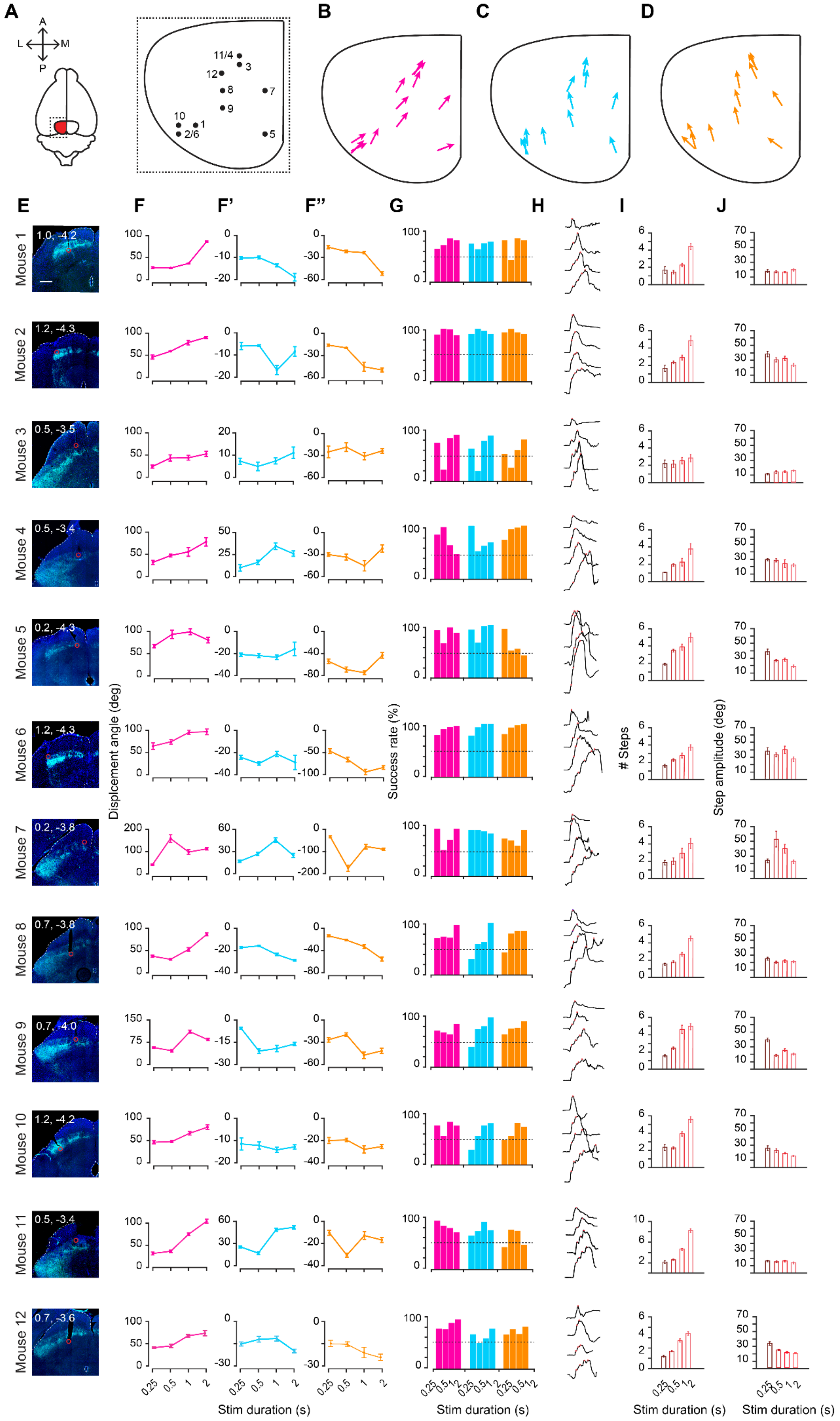

A
B
C
D
E
F
F'
F''
G
H
I
J
Mouse 1
Mouse 2
Mouse 3
Mouse 4
Mouse 5
Mouse 6
Mouse 7
Mouse 8
Mouse 9
Mouse 10
Mouse 11
Mouse 12
Displcement angle (deg)
Success rate (%)
Steps
Step amplitude (deg)
Stim duration (s)

Figure S6 │ Reliability of head kinematics driven by optical stimulation of Pitx2 neurons. Related to Figures 2 and 3.

A-D, Top view summary of implant locations (**A**) and summary of characteristic movement vector produced in yaw (**B**), pitch (**C**) and roll (**D**) for animals shown in **E-J**. **E**, Histology of the implantation site for each tested mouse with estimated implant coordinates (x, y; scale bar: 500 µm). **F-H**, Effect of stimulus duration on the head displacement angle (**F-F”**) and the success rate of a head movement bout (**G**) for each mouse for yaw (*magenta*), pitch (*blue*) and roll (*orange*). Kinematics of head displacements for each mouse along the yaw dimension for increasing duration of stimulation (0.25, 0.5, 1 and 2 s); note the presence of stepwise trajectories for prolonged stimulations (**H**). **I-J**, Trial-by trial average of the number of recorded steps (**I**) and average step amplitude (**J**) for each mouse for increasing stimulus duration (n_{MICE} = 12, $n_{TRIALS/MOUSE}$ = 60). All results are presented as mean ± SEM.

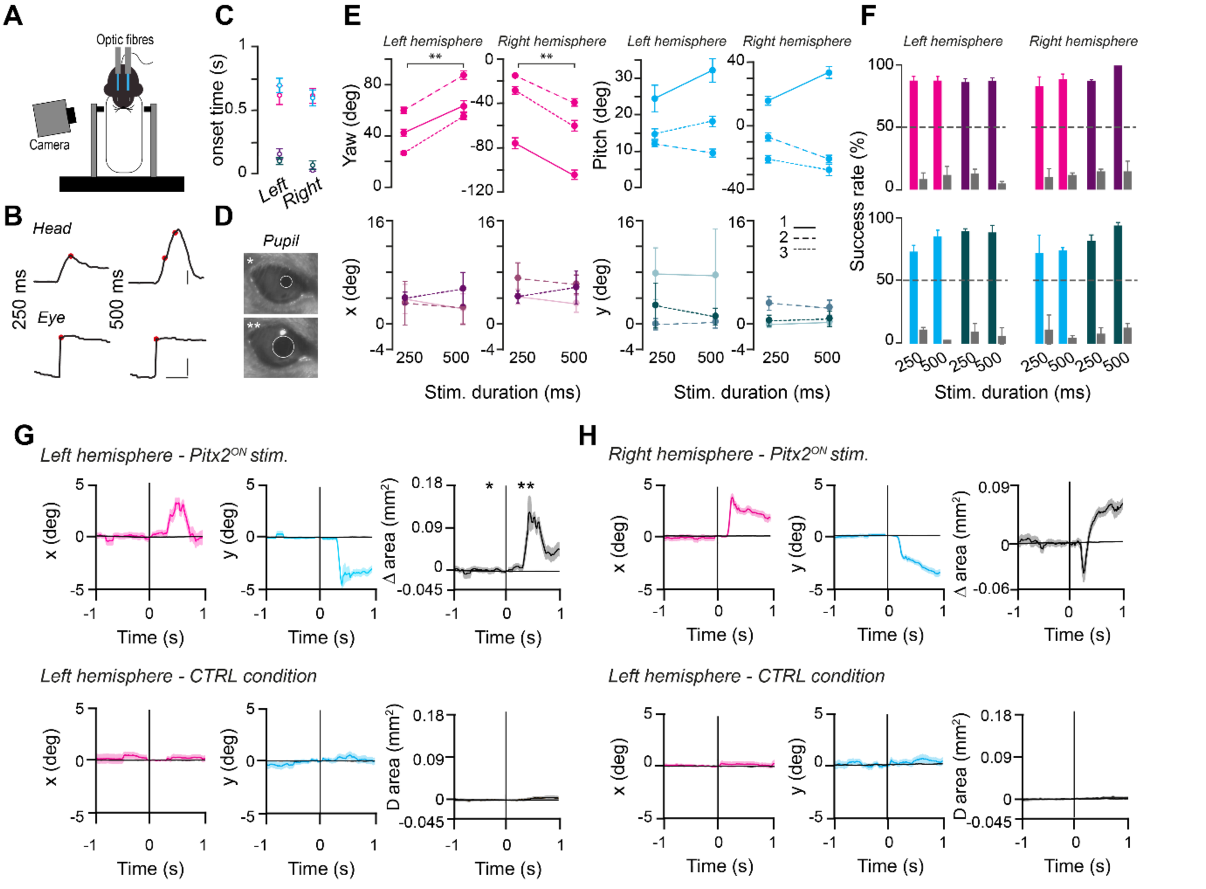

Figure S7 │ Eye and head movements elicited by optogenetic stimulations. Related to Figure 2.

A, Schematic of experimental setup for eye tracking in head restrained *Pitx2-CRE::Rosa-LSL-ChR2-eYFP* animals. Two optic fibres were implanted: one on the left and one on the right hemisphere to record movements evoked by a contra- or ipsi-lateral SC stimulation with respect to the monitored eye (right eye). **B**, Representative traces for yaw component for the head movement and x component for the eye movement after 250 (left) or 500 ms (right) of blue light stimulation of the left hemisphere. Red dots mark a change in velocity for the head and the eye motion (for head displacement, scale bars: 20 deg, 1 s; for eye movement, scale bars: 2 deg, 1 s). **C**, Time of onset of motion after blue light stimulation for yaw (*magenta*) and pitch (*blue*) and for x (*purple*) and y (*dark blue*) ($n_{MOUSE} = 3$; $n_{TRIALS/MOUSE} = 30$). **D**, Frames from the eye camera showing changes in pupil dilation (see panel **G**) taken before (*one asterisk*) and after (*two asterisks*) 250 ms blue light stimulation with fitted ellipses (*white*). **E**, Effect of stimulus duration on the head displacement angle for yaw and pitch (top), and on the eye movement for x and y after the stimulation of the left or right hemisphere ($n_{MOUSE} = 3$; $n_{TRIALS/MOUSE} = 30$; $p_{LEFT,YAW} = 0.008$, $p_{RIGHT,YAW} = 0.006$, $p_{LEFT,PITCH} = 0.9334$, $p_{RIGHT,PITCH} = 0.4251$, $p_{LEFT,X} = 0.11$, $p_{RIGHT,X} = 0.0712$, $p_{LEFT,Y} = 0.4367$, $p_{RIGHT,Y} = 0.72$). **F**, Effect of stimulus duration on the probability of executing a head movement bout in yaw and pitch, and eye movement in x and y after the stimulation of the left or right hemisphere. Grey bars for the effect of the stimulation in control mice in absence of ChR2 expression ($n_{MICE} = 3$, $n_{TRIALS/MOUSE} = 30$). **G**, Examples of light-triggered average (LTA) of eye movements along the x and y components, and of the pupil area (*grey*) calculated for blue light stimulation of 250 ms in the left hemisphere of the same animal in **B** and **D** expressing ChR2 in Pitx2^{ON} neurons (top) and in a control animal (bottom) ($n_{Pitx2\text{-}ChR2\ MICE} = 3$, $n_{CTRL\ MICE} = 3$, $n_{TRIALS/MOUSE} = 30$). **H**, Same as in **G** for the right hemisphere. All results are presented as mean ± SEM.
